# Genomic Epidemiology of ESBL and Non-ESBL-Producing *Escherichia coli* Across One Health Interfaces in Oman

**DOI:** 10.3390/antibiotics15040411

**Published:** 2026-04-17

**Authors:** Hibatallah Sultan Al-Habsi, Zaaima Al Jabri, Amina Al-Jardani, Amira ElBaradei, Hafidha Al-Hattali, Faiza Syed, Zakariya Al Muharrmi, Wafa Al Alawi, Hatim Ali Eltahir, Meher Rizvi

**Affiliations:** 1 Central Laboratory of Animal Health, Muscat 100, Oman; hibatallahalhabsi@gmail.com; 2 Department of Microbiology and Immunology, College of Medicine and Health Sciences, Sultan Qaboos University, Muscat 123, Oman; zaeema@squ.edu.om (Z.A.J.); a.elbaradei1@squ.edu.om (A.E.); 3 Central Public Health Laboratories, Center for Disease Control and Prevention, Ministry of Health, Muscat 113, Oman; aksaljardani@gmail.com; 4 Department of Biomedical Science, College of Medicine and Health Sciences, Sultan Qaboos University, Muscat 123, Oman; h.hattali@squ.edu.om; 5 DASH to Protect Antibiotics, Muscat 111, Oman; faizasy@gmail.com; 6 Department of Microbiology and Immunology, University Medical City, Muscat 112, Oman; almuharrmi@gmail.com; 7 Central Analytical and Applied Research Unit, Sultan Qaboos University, Muscat 123, Oman; wafaj@squ.edu.om; 8 Ministry of Agriculture and Fisheries, Muscat 100, Oman; hatem.ali@mafwr.gov.om

**Keywords:** One Health, *Escherichia coli*, ESBL, whole genome sequencing, human, animal, environment, AMR

## Abstract

**Background**: Antimicrobial resistance is a One Health problem driven by the intricate interactions across human, animal, and environmental interfaces that enable microbial exchange and movement of mobile genetic elements encoding resistance and virulence. This study investigated the genomic epidemiology of ESBL and non-ESBL *Escherichia coli* across One Health interfaces in Oman. **Methods**: This prospective cross-sectional study analyzed 295 non-duplicate *Escherichia coli* isolates derived from 104 clinical, 173 animal [diseased (123) and healthy (50)], 14 sewage and four water sources. Antimicrobial susceptibility testing was performed phenotypically, and a representative subset of 50 ESBL and non-ESBL *Escherichia coli* from the three interfaces underwent whole genome sequencing to determine MLST, phylogroups, resistance genes, virulence determinants and plasmid replicons. **Results**: ESBL prevalence was highest in human isolates (73%), followed by sewage (28.6%) and animals (16.3% diseased; 8% healthy). *bla*CTX-M-15 predominated in humans, whereas *bla*CTX-M-55 dominated in animals and sewage, suggesting ecological partitioning with partial overlap. Quinolone resistance was lowest in the animal interface. Sewage isolates harbored the most complex resistome, including *rmtB* and plasmid-mediated quinolone resistance genes. MLST analysis revealed high diversity in human isolates, including globally recognized ExPEC lineages (ST10, ST38, ST73, ST127, ST131), while ST224 dominated in animals with evidence of possible spillover to humans. ST167 was confined to sewage, consistent with environmental maintenance of high-risk clones. Phylogroup structuring showed predominance of A, B2 and D among human isolates and A, B1, and E among animal and sewage isolates. Virulence profiling demonstrated broader virulome diversity in humans, but shared core determinants (*fimH*, *sitA*, *traT*) across all domains. IncFIB(AP001918) was the dominant plasmid replicon, particularly among ESBL isolates, underscoring its role in horizontal gene dissemination. Alarmingly, mutation in *pmrB* (V161G) was identified in a healthy animal isolate, pointing to a need for greater colistin restriction in animal husbandry. **Conclusions**: This study highlights plasmid-mediated resistance and shared virulence determinants linking reservoirs; although AMR profile was quite distinct across the three interfaces, human isolates demonstrated greater resistance than animal isolates, suggesting healthcare-driven AMR in Oman. Continued integrated genomic surveillance is essential to monitor gene flow and inform coordinated antimicrobial stewardship strategies.

## 1. Introduction

The growing recognition of the interconnectedness between human, animal, and environmental health has made the assessment of antimicrobial resistance (AMR) within the One Health framework exceedingly important. AMR is a quintessential One Health problem, with the intricate integration of microbial exchange across the three interfaces allowing seamless movement of mobile genetic elements encoding resistance and virulence [1]. This global problem impacts not just human health but also animal health, food production, and environmental conditions [2].

Although many countries have drafted national action plans, implementation and dedicated budgets remain limited, underscoring the gap that One Health approaches aim to close. To narrow this gap, the World Health Organization (WHO) has introduced the Tricycle project which promotes multisectoral surveillance of Extended-Spectrum Beta-Lactamase (ESBL) carrying *Escherichia coli* [3].

*E. coli* is an ideal sentinel across the three interfaces: it is ubiquitous, easily cultured from clinical, veterinary, and environmental matrices, and frequently harbors plasmid-borne ESBL enzymes, like CTX-M/SHV/TEM. CTX-M alleles display setting-specific distributions—CTX-M-15 in human clinical isolates versus CTX-M-55 in bovine and wastewater sources—reflecting ecological selection and reservoirs. Increasingly, food-producing animals are being considered sources of ESBL *E. coli* in humans; Ludden et al. have reported limited evidence linking AMR in human-derived *E. coli* to livestock in their region [4,5,6].

Virulence genes are central to the disease-causing capacity of microbial pathogens, as they encode factors that enable organisms to adhere to hosts, establish colonization, invade tissues, evade immune defenses, and produce clinical manifestations of infection [7]. Increasing evidence indicates that virulence determinants frequently co-evolve and co-disseminate with antimicrobial resistance genes, particularly when they are carried on mobile genetic elements such as plasmids and transposons [8]. In the pandemic ST131 *E. coli*, the concurrence of virulence traits and antimicrobial resistance has been documented, indicating an alarming genetic convergence of virulence and resistance within this globally successful clone [9].

This study from Oman was a coordinated effort at sampling ESBL and non-ESBL *E. coli* from clinical sites, diseased and healthy animals, sewage and water systems (falaj/wells), enabling side-by-side comparison of phenotype, genotype, and plasmid ecology. Whole genome sequencing (WGS) was carried out to assess and compare sequence types, resistance and virulence genes carriage, and plasmids, aligning our study with One Health priorities: mapping gene flow among animals, humans, and the environment to quantify risks of transfer and to guide surveillance, stewardship, and WASH interventions.

## 2. Results

### 2.1. Antimicrobial Susceptibility Profile Across the Three Domains

**Figure 1:**
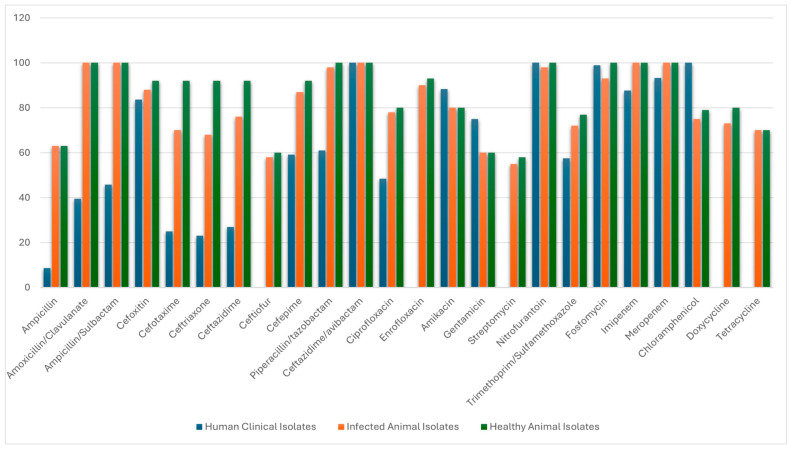
Antimicrobial susceptibility profile of *Escherichia coli* from human and animal clinical isolates and from healthy animals.

The *E. coli* isolates from humans carried the highest burden of ESBLs (73%, 76/104), followed by sewage (28.6%, 10/35) and diseased animals (16.3%, 20/123), while no ESBLs were detected in drinking water. AmpC carriage was highest in human isolates (11.5%, 12/104) while it was half that in diseased animals (6.5%, 8/123), with none being detected in sewage or drinking water samples. CRE was restricted only to human isolates (4.8%, 5/104) and was absent in animals, sewage, and drinking water. No ESBLs, AmpC or CRE were found in healthy animal samples and water (Supplementary Table S1). A detailed susceptibility profile of human and animal isolates is shown in Figure 1. As expected, susceptibility to third and fourth generation cephalosporins, and beta lactam-beta lactamase inhibitors, were significantly lower in human isolates compared to animal isolates; the average third and fourth generation cephalosporin rates were 25% (26/104) and 59% (61/104) in the former, while it was 71% (87/123) and 87% (107/123) respectively in the latter. Significantly, lower rates were observed in amoxicillin-clavulanic acid (39%, 41/104) and piperacillin-tazobactam (61%, 63/104) in human isolates, while all healthy and diseased animal isolates were susceptible. In contrast, higher susceptibility to aminoglycosides were noted in human isolates (88%, 92/104 for amikacin; 75%, 78/104 for gentamicin) compared to animals (80%, 98/123 for amikacin; 60%, 74/123 for gentamicin), with streptomycin rates being lowest at 55%, 68/123 in animals. Surprisingly, enrofloxacin demonstrated superior rates than ciprofloxacin in animal isolates (90%, 110/123 and 93%, 114/123 in diseased and healthy isolates, respectively). Chloramphenicol demonstrated 100% susceptibility against human isolates compared to 75% (92/123) against diseased animals.

The susceptibility rates of beta lactam antimicrobials were significantly lower in human isolates compared to the animal isolates (Table 1); the average third and fourth generation cephalosporin rates were 25% and 59% in the former, and 71% and 87% respectively in the latter. Similarly, lower rates were observed against beta-lactam-beta-lactam inhibitors in human isolates (39% for amoxicillin, clavulanic acid, and 61% for piperacillin-tazobactam), while 100% rates were observed in both healthy and diseased animal isolates. Fluoroquinolones demonstrated similar results. Surprisingly, enrofloxacin demonstrated superior rates than ciprofloxacin in animal isolates (90% and 93% in diseased and healthy isolates). In contrast, the reverse was true for aminoglycosides. Higher rates were noted in human isolates (88% amikacin, 75% gentamicin), and lower rates (80% amikacin, 60% gentamicin) in animals. Streptomycin susceptibility was even lower: 55% and 58% in diseased and healthy animals. Surprisingly, chloramphenicol demonstrated 100% susceptibility against human isolates compared to 75% and 79% against diseased and healthy animals, respectively.

### 2.2. Distribution of AMR Genes and Mutations

**Figure 2:**
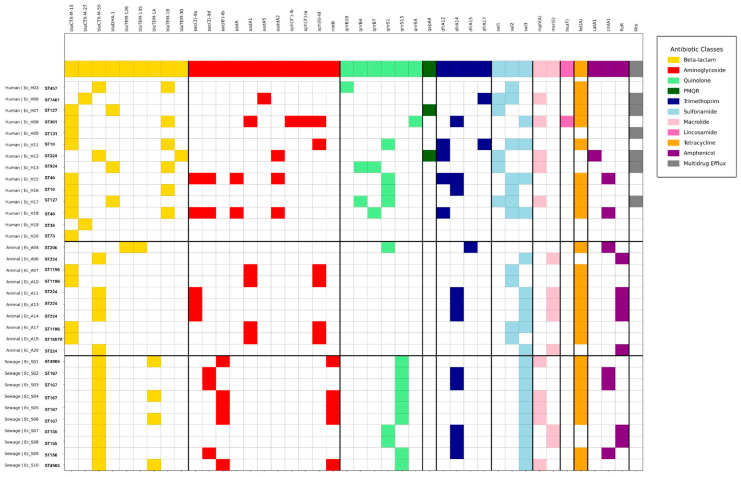
Heatmap depicting distribution of AMR genes across the three One Health interfaces. In humans the prevalence of *bla*CTX-M-15 was the highest (9/14), followed by 2/14 each of *bla*CTX-M-27 and *bla*CTX-M-55. *bla*CTX-M-55 was found in 9/10 animal isolates; 5/10 animal isolates carried *bla*CTX-M-55 and 4/10 had *bla*CTX-M-15. In the sewage samples, all the isolates carried *bla*CTX-M-55.

Amongst the representative ESBL isolates sent for WGS, β-lactamase gene distribution differed across the domains (Figure 2). In humans, *bla*CTX-M-15 (9/14) dominated, followed by 2/14 each of *bla*CTX-M-27 and *bla*CTX-M-55, while among 9/10 animal isolates, five carried *bla*CTX-M-55 and four *bla*CTX-M-15, and all sewage isolates carried only *bla*CTX-M-55. *bla*DHA-1 (AmpC) was seen in only three human isolates, co-harbored with *bla*CTX-M-15. Narrow-spectrum *bla*TEM-1B was co-carried in 6/14 human isolates and *bla*TEM-1A in 4/10 in sewage.

Diverse aminoglycoside resistance genes were detected across the interfaces: acetyltransferase genes (*aac*(3)-*IIa*, *aac*(3)-*IId*, *aac*(6)-*Ib)*, adenylyltransferase genes (*aadA*1, *aadA*2, *aadA*5), phosphotransferase genes (*aph*(3′)-*Ib*, *aph*(6)-*Id*), and the 16S rRNA methyltransferase gene *rmtA*. Animal isolates demonstrated greater aminoglycoside resistance, with 7/10 carrying one or two resistance genes; three carried *aac*(3)-*IIa* consistent with gentamicin, tobramycin and netilmicin resistance and four co-harbored *aadA* and *aph*(6)-*Id* consistent with streptomycin and kanamycin resistance. Sewage isolates displayed the most complex aminoglycoside resistome, with 5/10 co-carrying *rmtB*, which leads to pan aminoglycoside resistance, and *aac*(6)-*Ib* (amikacin, tobramycin and netilmicin resistance). Less resistance was observed in humans. Two isolates co-harbored *aac*(3)-*IIa*, *aac*(3)-*IId*, *aadA*1 and *aadA*2 and one co-carried *aadA*1, *aph*(3′)-*Ia*, *aph*(3′)-*Ib*, *aph*(3′)-*Id* and *aph*(6)-*Id*.

Quinolone resistance genes were observed in 8/14 humans, all sewage isolates and one animal isolate. Plasmid-mediated quinolone resistance (PMQR) genes predominated [*qnrS*1 (4/14)] in humans, while 8/10 sewage isolates carried *qnrS*13, with the rest carrying *qnrS*1.

Mutations conferring resistance to ciprofloxacin and nalidixic acid were detected in 28 isolates (eight from human clinical, 10 from animals and 10 from sewage), while mutations conferring resistance to colistin were found in only one isolate from animals (Table 1). All 28 isolates had *gyrA* mutations, *parC* in 26 and *parE* in 22 isolates. Among human isolates, six co-harbored *gyrA*, *parC* and *parE* mutations, while two had mutations only in *gyrA*. In animal isolates, nine co-harbored *gyrA*, *parC* and *parE* mutations and one co-harbored *gyrA* and *parC* mutations. Seven sewage isolates co-harbored *gyrA*, *parC* and *parE* mutations and three isolates *gyrA* and *parC* mutations. Interestingly, a mutation in *pmrB* (V161G) was found in an healthy animal.

A wider spectrum of trimethoprim resistance genes (*dfrA*12, *dfrA*14, *dfrA*17) were identified in human isolates, while *dfrA*14 predominated in animal and sewage isolates. Sulfonamide resistance genes were among the most broadly distributed resistance determinants, with *sul*1, *sul*2, *and sul*3 being equally represented in humans, *sul*2 and *sul*3 in animals, and *sul*3 in sewage isolates. The macrolide resistance gene *mph*(*A*) was detected in human and sewage isolates, the *mrx* gene exclusively in humans and *msr*(*E*) in animal isolates. Barring a few isolates, the tetracycline resistance gene *tet*(*A*) was present in all three domains. Amphenicol resistance genes (*cmlA*1, *floR*) were detected predominantly among animal and sewage isolates. One human isolate carried *catA*1 and two *cmlA*1.

A narrow repertoire of antimicrobial resistance genes was detected among non-ESBL isolates compared to ESBL isolates. Although resistance nodulation cell division (RND) efflux pump components were widely distributed across both ESBL and non-ESBL *E. coli*, ESBL isolates demonstrated a higher prevalence of efflux regulators and auxiliary efflux systems, including *marA*, *emrB*, *emrR*, *emrK* and *emrY* (Supplementary Table S2). For sulfonamide, trimethoprim, macrolides and quinolone resistance genes, only *sul*1, *dfrA*17, mrx and qnrB were detected in non-ESBL clinical isolates—these were absent in non-ESBL animal isolates. However, non-ESBL animal isolates showed more diversity in RND genes and major facilitator superfamily (MFS) genes compared to human non-ESBL isolates.

### 2.3. MLST Distribution

**Figure 3:**
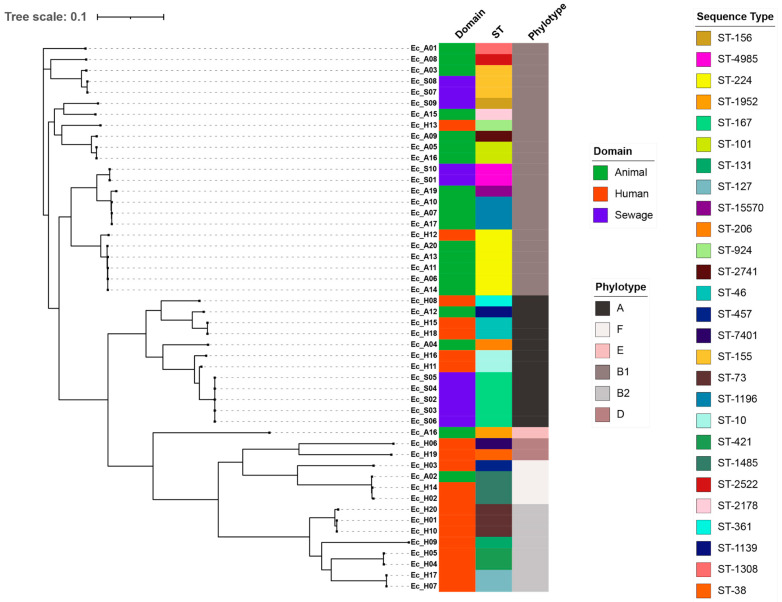
Phylotype structure and ecological distribution. No distinct MLST pattern was observed across the three interfaces (human, animal and environment). The highest diversity was observed in human isolates, with 13 STs altogether from 11 ESBL and three non-ESBL isolates. Four STs were found to be clustered in animal and sewage isolates (ESBL producers). There was no distinction of lineages between ESBL and non-ESBL *E. coli*.

MLST distribution of *E. coli* was explored to assess convergence of transmission pathways across clinical, veterinary and environmental reservoirs (Figure 3, Table 2). No clear MLST pattern was observed across the three interfaces, with the human isolates demonstrating the greatest diversity (13 STs overall; 11 in ESBL and three in non-ESBL, with ST73 present in both). Clustering of four STs each were observed in animal and sewage ESBL isolates, with nine additional STs observed in non-ESBL animal isolates. There was a clear separation of lineages between ESBL and non-ESBL *E. coli*, with occasional crossovers (ST73 and ST155).

Overall, the ESBL population was characterized by 18 ST types. ST224 and ST1196 dominated in animals and ST167, ST155, and ST4985 in sewage, while no striking pattern was noted in humans (although ST10, ST46 and ST127 featured in two each). Surprisingly, only one human isolate carried the global multidrug resistant clone ST131. A possible spillover of *bla*CTX-M-55 carrying ST224 was observed from animals to humans and non-ESBL ST155 from the environment to animals (one isolate each). The animal ST224 was characterized by the same resistance genes. In human isolates, ST38 and ST73 were remarkable for not carrying any resistance genes other than *bla*CTX-M27 and *bla*CTX-M15.

The non-ESBL isolates were categorized into 11 STs of which only three STs (ST73, ST421 and 1485) were identified amongst human isolates. No distinct pattern was observed among animal non-ESBL *E. coli* isolates, with only one ST (ST101) occurring twice.

### 2.4. Phylotype Structure and Ecological Distribution

Phylotyping revealed a clear pattern, with isolates spread over phylotypes A, B1, B2, D, E and F (Figure 3). Human isolates predominated in A, B2 and D. They formed distinct clades on the tree, representing specialized pathogenic lineages. In contrast, animal and sewage isolates were more frequently associated with phylotypes A, B1, and E, which are commonly linked to commensal strains and environmental persistence. Phylotype F isolates were less common but were observed within clusters containing both human and environmental isolates.

### 2.5. Serotyping

The in silico analysis revealed distinct, partially overlapping serotype distributions, although complete overlap at the O:H serotype level was limited (Table 3). Human isolates exhibited broader serotype diversity with O6 and O9 being the predominant variants, each comprising 25% (5/20) of the isolates. No significant H-antigen predominance was observed, with H1 being most frequent (15%, 3/20) while H42, H7, H9, H18, H30, and H10 each represented 10% of isolates. A contrasting pattern was observed in animal isolates, with 30% (6/20) of O-antigens remaining untyped, with O78 dominating (25%, 5/20) followed by H23 (25%, 5/20) and H10 (20%, 4/20). Sewage samples demonstrated markedly lower diversity, with O101 and H10 representing 50% (5/10) each. Notably, the O25:H4 serotype was detected in human isolate EcH09 (CTX-M-15 positive) and animal isolate EcA12 (O9:H4, non-ESBL producer), indicating that they may potentially be related.

### 2.6. Virulome Repertoire

Virulome profiling of ESBL-producing *E. coli* revealed marked differences in distribution across the three interfaces (Table 4) although several virulence factors like curli production (*csgA*), type 1 fimbrial adhesion (*fimH*), serum resistance (*traT*), survival (*terC*) outer membrane protease (*ompT*), and key iron acquisition systems (*iroN*, *iucC*, *iutA*, *sitA*) were shared across human, animal, and sewage isolates.

Human-derived isolates demonstrated a wider diversity of virulence-associated genes (41 genes), with the following groups dominating: adhesion and colonization (*afaA*, *sfa*, *foc*, *fim*, *iha*, *pap*, and *tia*), immune evasion (*kpsE*, *kpsMII*, *iss*, *traT*, and *ompT*), iron acquisition (*chuA*, *fyuA*, *iroN*, *irp*2, *iucC*, *iutA*, *sitA*), and toxicity (*cnf*1, *hlyA*, *hlyF*, *hlyE*, and *usp*). Animal-derived isolates exhibiting fewer genes (16 genes) shared the same iron uptake systems (*iroN*, *iucC*, *iutA*, and *sitA*) and serum resistance proteins (*traT*, *ompT*). Sewage isolates carried 24 virulence genes, some common with both human or animal sources like adhesins (*csgA*, *fimH*, *ipfA*, *papC*), while *tia*, *yehC*, *and yehD* were seen exclusively in sewage isolates. Both human- and sewage-derived isolates carried *fyuA*, *hlyE* and *hlyF*. Strains from animals and sewage shared *astA*, *cib*, *and cvaC*.

On analyzing genes present in more than 50% of ESBL isolates across the three domains, *fimH* (57.1%), *gadA* (71.4%), *chuA* (50.0%), *fyuA* (57.1%), and *sitA* (71.4%) predominated in human isolates. Animal-derived *E. coli* was characterized by the universal presence of *fimH*, *traT*, *iucC*, and *sitA,* and high prevalence of *ompT* (90%), *iutA* (60%), and bacteriocin genes (*cea*, *cma*, *cvaC*). All sewage isolates carried *fimH* and the majority carried *sitA* (80%), *traT* (80%), *terC* (70%), *cia* (90%), *cvaC* (80%), *cma* (60%), and *astA* (70%). Overall, across the three domains, only *fimH*, *sitA* (both 82.4%) and *traT* (67.6%), were carried by majority isolates.

### 2.7. Plasmid Distribution

**Figure 4:**
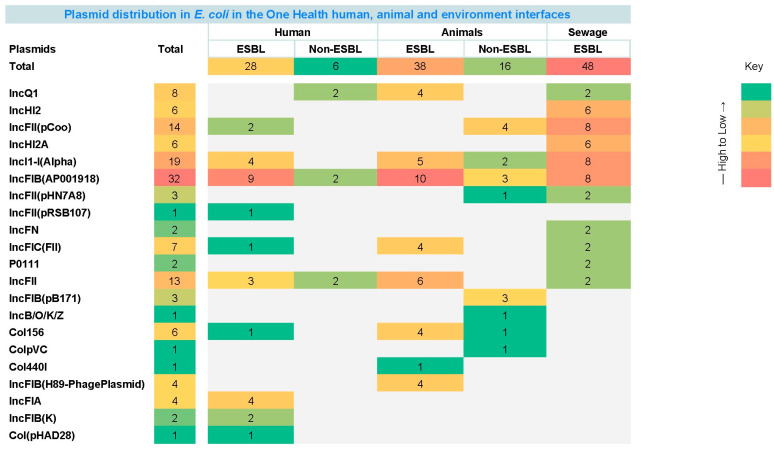
Comparative analysis of Plasmid distribution in the three interfaces. Among the ESBL and non-ESBL isolates, 21 distinct plasmid replicon types were detected from human (11), animal (13) and sewage samples (11).

Overall, a total of 21 different plasmid replicon types were detected across the three domains (humans (11), animals (13), sewage (11)) in ESBL and non-ESBL isolates. Amongst these, IncFIB(AP001918) was the most prevalent plasmid replicon type (32/50, 64%), while IncFII(pRSB107), IncB/O/K/Z, ColpVC, Col440I and Col(pHAD28) were the least detected, each being detected once (1/50, 2%) (Figure 4).

In human ESBL isolates (14), the most frequently detected plasmid replicon type was IncFIB(AP001918) (9/14, 64.29%), followed by Incl1-1(Alpha) and IncFIA (4/14, 28.57% each). However, among non-ESBL isolates (6), only three plasmid replicon types were detected with identical frequency (2/6, 33.33% each).

Similarly, in animal ESBL isolates, the most frequently detected plasmid replicon type was also IncFIB(AP001918) (10/10, 100%), followed by IncFII (6/10, 60%), Incl 1-1(Alpha) (5/10, 50%), and then Col156, IncFIB(H89-PhagePlasmid), IncFIC(FII) and IncQ1 (4/10, 40% each). Animal non-ESBL isolates (10) carried a wider portfolio of plasmids (8) compared to human non-ESBLs (3). The most detected plasmid replicon type among them was IncFII(pCoo) (4/10, 40%), followed by IncFIB(AP001918) and (IncFIB(pB171) (3/10, 30% each), then Incl 1-1(Alpha) (2/10, 20%).

Among all domains, the highest number of plasmid replicon types was detected in sewage isolates. The most frequently detected plasmid replicon types in sewage isolates were IncFIB(AP001918), Incl 1-1(Alpha) and IncFII(pCoo), which were detected among 8/10, 80% each, followed by IncHI2 and IncHI2A (6/10, 60% each). The least detected plasmid replicon types were IncQ1, IncFII(pHN7A8), IncFN, IncFIC(FII), P0111 and IncFII (2/10, 20% each).

## 3. Discussion

A One Health approach provides valuable insights into the dynamic interconnections between human, animal, and environmental reservoirs that impact the distribution of AMR, virulence traits, phylogenetic lineages, and mobile genetic elements. This study on One Health from Oman yielded many interesting insights.

The significantly higher burden of ESBL, AmpC and CPE in human *E. coli* in our study is noteworthy and consistent with global surveillance data (https://www.who.int/initiatives/glass, accessed on 18 December 2025). The absence of CPE in the other two interfaces suggests that carbapenem resistance in Oman is healthcare-driven while aminoglycoside and chloramphenicol resistance reflects veterinary selection pressures. While *bla*CTX M-55 was common in all three interfaces, human isolates also carried *bla*CTX M-15, *bla*CTX M-27, and animals *bla*CTX-M15, while sewage only carried *bla*CTX-M-55, suggesting greater horizontal gene transmission between the latter two. Dominance of *bla*CTX-M15 in human isolates is consistent with its global presence in clinical ExPEC infections [10,11]. The carriage of only *bla*CTX-M55 in sewage *E. coli* and its dominance in animal isolates aligns with reports on the increasing association with livestock, food chains, and environmental dissemination [12]. However, Yu et al. have reported a predominance of CTX-M55 in clinical isolates [13].

The presence of pan aminoglycoside resistance determinants, 16S rRNA methyltransferase *rmtB* and *aac*(6′)-*Ib* in sewage is extremely worrisome. Zhang et al. have reported similar findings in wastewater systems [14,15]. It is particularly concerning because they are frequently plasmid-borne and co-localize with ESBL genes, facilitating co-selection and persistence even when aminoglycoside exposure is intermittent [15].

A distinct PMQR gene distribution was observed with all sewage isolates carrying exclusively *qnrS*13 (80%) or *qnrS*1(20%); animals stood out with the least carriage while human isolates carried a varied set, though *qnrS*1 predominated. Banjo et al. reported higher prevalence of *qnrA* in hospital wastewater [16]. These findings support distinct pathways of dissemination of mobile quinolone resistance and point towards stepwise evolution toward high-level fluoroquinolone resistance [17,18]. The widespread presence of trimethoprim and sulfonamide resistance genes (*dfrA* variants and *sul*1/*sul*2/*sul*3) across domains reflects not only sustained antimicrobial exposure in both community and agricultural settings but also their association with mobile genetic elements [19]. Interestingly, there was blanket carriage of *tet*(*A*) across all domains, while amphenicol resistance genes (*floR*, *cmlA*1) were seen commonly in animal and sewage isolates—findings consistent with livestock-associated selection pressures and downstream environmental release. It is important to note that *floR* and *cmlA* are often found to be co-located with *tet* genes on the same plasmids [20].

Fluoroquinolone mutations (*gyrA*, *parC* and *parE)* predominated in sewage isolates. Both human and animal isolates predominantly demonstrated mutations in all three genes. Point mutations were detected in the quinolone resistance-determining region (QRDR) of the DNA gyrase (g*yrA*) as well as the DNA topoisomerase IV (*parC*) and (*parE*). However, no mutations were detected in (g*yrB*) DNA gyrase, a finding corroborated by previous studies [21,22].

This expanded efflux repertoire in ESBL strains compared to non-ESBL strains suggests enhanced possession of intrinsic resistance mechanisms that may act synergistically with β-lactamase production to promote multidrug resistance and persistence under antimicrobial pressure [23]. The similar distribution of efflux-associated genes among human and animal non-ESBL *E. coli* isolates suggests conservation of these across reservoirs.

In this study, ST1952 isolated from a healthy animal from Muscat Governorate harbored a *pmrB* mutation. To the best of our knowledge, this is the first report of *pmrB* mutation conferring colistin resistance from Oman in animals. This may be attributed to the use of colistin not only in veterinary medicine to treat infections, but also to its use as a growth promoter additive in livestock feed, which contributes to the presence of residues of the antibiotic in the fecal matter, hence contributing to the spread of colistin resistance in the surroundings [24]. Aworh et al. [22] reported a similar finding (V161G) from chicken in a poultry farm. Mutations in the *pmrAB* two-component regulatory system cause overexpression of certain bacterial operons (*pmrHFIJKLM* and *pmrCAB*), which in turn results in colistin resistance by modification of the LPS structure [25].

Several important MLST lineages were identified (ST10, ST38, ST46, ST73, ST127, ST131, ST361, 155), many of which belong to well-described extraintestinal pathogenic *E. coli* (ExPEC) lineages associated with urinary tract and bloodstream infections in humans (e.g., ST131, ST73) or with food, animal and environmental reservoirs (e.g., ST10 complex, ST155) [26]. Co-existence of such globally recognized “high-risk” STs with more niche-specific STs (such as ST7401 or ST5713 in humans, ST1196 in animals and ST4985 in sewage) underscore the genomic plasticity of *E. coli* and the likelihood of both clonal expansion and horizontal gene transfer [27]. Overall, isolates within the same ST and interface carried the same resistance genes [28].

ST224 is an international high-risk clone often associated with *bla*CTX-M variants (*bla*CTX-M-15 and *bla*CTX-M-55) in poultry and human isolates [28]. This finding strengthens the possibility of food-producing animals contributing ESBL-producing *E. coli* to humans either via direct contact, food chains or shared environmental sources [29]. However, it was noted in our study that the human ST224 had AMR genes distinct to that of animals’ ST224. ST167 carrying *bla*CTX-M-55 and *rmtB* dominated and were restricted to sewage isolates, suggesting that it may primarily be maintained in environmental or mixed human waste reservoirs rather than in the sampled animal or clinical populations. However, Mujahid et al. has reported it in uropathogenic human isolates [30]. It belongs to the ST10 clonal complex, which is widely recognized as a host-generalist One Health lineage found in humans, animals and environmental niches, frequently carrying *bla*CTX-M-15, other ESBLs and sometimes mcr-1 and carbapenemase genes [30]. The confinement of ST167 to sewage in our dataset is consistent with recent wastewater studies that demonstrate high diversity of ESBL-producing *E. coli* with over-representation of globally prevalent lineages such as the ST10 complex, ST38, ST69 and ST131 in wastewater and surface waters [31,32]. ST167 *E. coli* carrying *bla*CTX-M-55 and *bla*NDM-5 have been isolated from public environments, including municipal sewage, indicating potential waterborne transmission risks [32]. This supports the role of sewage as a mixing hub and amplifier of high-risk clones originating from multiple upstream sources.

The clear distinction between STs in non-ESBL and ESBL human and animal isolates suggests that they belong to distinct lineages. ST1485 and ST421 were largely confined to non-ESBL human isolates, and ST101 and several ST types in animal isolates, indicating circulation of specific low-resistance or commensal lineages in the community [33]. Notably, ST1485 has recently been recognized as a globally disseminated, high-risk, phylogroup F clone with zoonotic potential, frequently carrying ColV plasmids and multidrug resistance determinants [34,35]. Its presence here as a non-ESBL type suggests its potential as a reservoir for acquiring ESBL and additional resistance genes over time, underlining the need for continued surveillance of apparently “susceptible” community lineages. Taken together, the ST patterns align with recent One Health genomic studies which show only partial overlap between human and livestock ESBL-producing *E. coli* populations, with transmission often mediated by shared plasmids and mobile genetic elements rather than wholesale sharing of identical clones [6,36].

Phylotyping demonstrated clear ecological structuring, with human isolates clustering predominantly within phylogroups A, B2 and D, the latter two being classically associated with extraintestinal pathogenic *E. coli* (ExPEC) lineages and enhanced virulence potential [37]. Phylogroups A, B1 and E in animal and sewage isolates are commonly linked to commensal populations and environmental persistence, reflecting their broader ecological plasticity [38]. The presence of phylogroup F within mixed human–environmental clusters suggest potential cross-domain transmission, a pattern increasingly recognized in One Health genomic surveillance studies [6,26].

Although human and animal domains exhibited a dominant serotype signature, the detection of O83:H42 in both domains suggests possible shared reservoirs. However, the limited number of identical serotype combinations indicates that direct cross-domain transmission may be less frequent than anticipated. Recent Australian One Health surveillance [39] has demonstrated that phylogenetic linkages at ≤100 SNP thresholds enable more accurate detection of cross-source transmission than serotyping alone. Although H10 was detected across all three domains, it was dominant in sewage. The ubiquitous presence of H10 across human, animal, and sewage domains aligns with Watt et al.’s (2025) demonstration of environmental compartments as key for *E. coli* lineage circulation in One Health surveillance [39]. The detection of O25:H4 (Ec_H09) is epidemiologically important. O25:H4 is strongly associated with the global multidrug-resistant ST131 lineage, a pandemic ExPEC clone responsible for urinary tract and bloodstream infections worldwide [40,41]. The ESBL-producing isolate of O25:H4/ST131 clonal group (Ec_H09) were CTX-M-15-producing. *E. coli* O25:H4/ST131 CTX-M-15-producing isolates have been reported in other countries [42].

The virulome patterns identified across human, animal, and sewage isolates underscore the multifaceted nature of ESBL-producing *E. coli* as a critical One Health pathogen [36]. The detection of core virulence determinants (*fimH*, *sitA*, *traT*) across all three interfaces supports the idea that *E. coli* maintains a conserved set of adhesins, iron uptake systems, and serum resistance genes, reflecting their functional importance in diverse niches [43,44]. Abeni et al. have also reported the ubiquitous presence of *fimH*, pointing to its evolutionary utility both within and across the interfaces [45]. Iron acquisition genes serve a valuable role in pathogenicity, with *sitA*, *iucC*, *iutA*, and *iroN* widely distributed across interfaces, supporting the growing evidence that these genes are central to the success of multidrug-resistant *E. coli* [46]. The presence of all eight genes in the human isolates reflects the critical nature of iron scavenging systems in establishing ExPEC fitness, ensuring survival in iron-limited as well as nutrient-poor environmental settings, while the near universal presence of *sitA* underscores its role in establishing *E. coli’s* versatility, findings corroborated by Gagaletsios et al. [47].

Human isolates possessed a wider virulome spectra (multiple adhesins, capsules, toxins, immune evasion/survival genes and siderophore systems) compared to animal and environmental isolates, pointing to greater selective pressures. One Health studies demonstrate that virulence gene diversity and abundance correlate with increased resistance burdens and clinical severity, further emphasizing the intertwined nature of virulence and multidrug resistance in extraintestinal *E. coli* [43]. Animal-derived isolates demonstrated high prevalence of serum resistance factors (*traT*, *ompT*) and aerobactin-mediated iron acquisition (*iucC*, *iutA*), enhancing survival in bloodstream and systemic infections. These are strongly associated with ExPEC plasmids, raising concerns of livestock becoming potential reservoirs of virulence–resistance plasmids, enhancing the potential of zoonotic transmission [26].

Sewage and animal isolates carried largely common bacteriocins (*cea*, *cib*, *cma*, *cvaC*) and immune evasion/survival genes (*ompT*, *traT*, *terC*), with the bacteriocins being more abundant in the former while the reverse was largely true for the latter. The wastewater virulome reflects the intense microbial competition with bacteriocins providing a competitive advantage. The presence of this cocktail of genes alongside classical ExPEC determinants such as *fimH* and *traT* suggests the pivotal role wastewater systems may play in potentially reshuffling virulence and resistance traits via horizontal gene transfer [48,49].

In our study, IncFIB(AP001918), a conjugative plasmid replicon type, was the most prevalent among the 21 plasmids across the domains in ESBL *E. coli*, being present in all animal isolates, 80% of sewage and 64.29% of clinical isolates. Its dominant presence poses a grave threat as transmission of AMR and virulence genes across the three domains can be easily facilitated via horizontal transfer. IncFIB(AP001918) plasmids are highly stable, broad-host-range, and frequently carry ESBL genes (*bla*CTX-M-15), quinolone resistance (qnr), and virulence factors (e.g., siderophores), enabling One Health transmission [50,51,52]. IncFIB(AP001918) facilitates AMR spread from livestock/poultry to humans through food and the environment [53].

Interestingly, the non-ESBL animal isolates carried a larger repertoire of plasmids compared to human non-ESBL isolates. Some were unique to non-ESBL animal isolates, like IncFII(pHN7A8), IncFIB(pB171), IncFII(pCoo) and IncB/O/K/Z. Not many studies have compared plasmids in non-ESBL animal and human isolates. Animal commensal *E. coli* inhabit more diverse ecological niches, like shared housing, feed, water, and environmental interfaces, which enhance opportunities for horizontal plasmid acquisition [54]. Livestock-associated E. coli frequently harbor IncF-, IncI1-, and ColV-like plasmids that may carry fitness, colonization, iron-acquisition, or bacteriocin-associated traits rather than ESBL genes alone, allowing persistence [55]. The fewer plasmids in human non-ESBL clinical isolates may be attributed to the metabolic cost entailed in maintaining non-essential ones in the face of selective pressure imposed by antibiotic exposure.

## 4. Materials and Methods

This prospective, cross-sectional collaborative One Health study, conducted from September 2023 to November 2024, characterized representative ESBL and non-ESBL *E. coli* across the three ecological interfaces (human, animal, and environment) in the Sultanate of Oman. The Department of Microbiology and Immunology, Sultan Qaboos University, collaborated with Sultan Qaboos University Hospital, the Central Laboratory of Animal Health (CLAH), and the Central Public Health Laboratory at the Ministry of Health and NAMA Water Services. Ethical approval was obtained from the Medical Research Ethics Committee (MREC) at the College of Medicine & Health Sciences, SQU (REF.NO.SQU/EC/2678).

### 4.1. Sample Collection and Processing

Consecutive *E. coli* isolates from clinical, animal sources, water, and sewage sources were included in the study. Sample size calculation for animal, human, and environmental samples was performed using the ANOVA table since more than two groups were analyzed. Based on insights from prior research, it was established that the *E. coli* mean effect size was 0.330 with a 5% margin of error and a 95% confidence level [56]. The mean effect size was 0.33, confidence interval = 95%, degree of freedom 2, 90% power, and α = 5%. The resulting calculated sample size was determined to be approximately 48 samples from each group. However, in anticipation of potential missing data, the sample size was increased to 100 from each source to enhance the robustness of the study’s findings. Details of sample collection for water and sewage samples is provided in Supplementary File S1.

**Figure 5:**
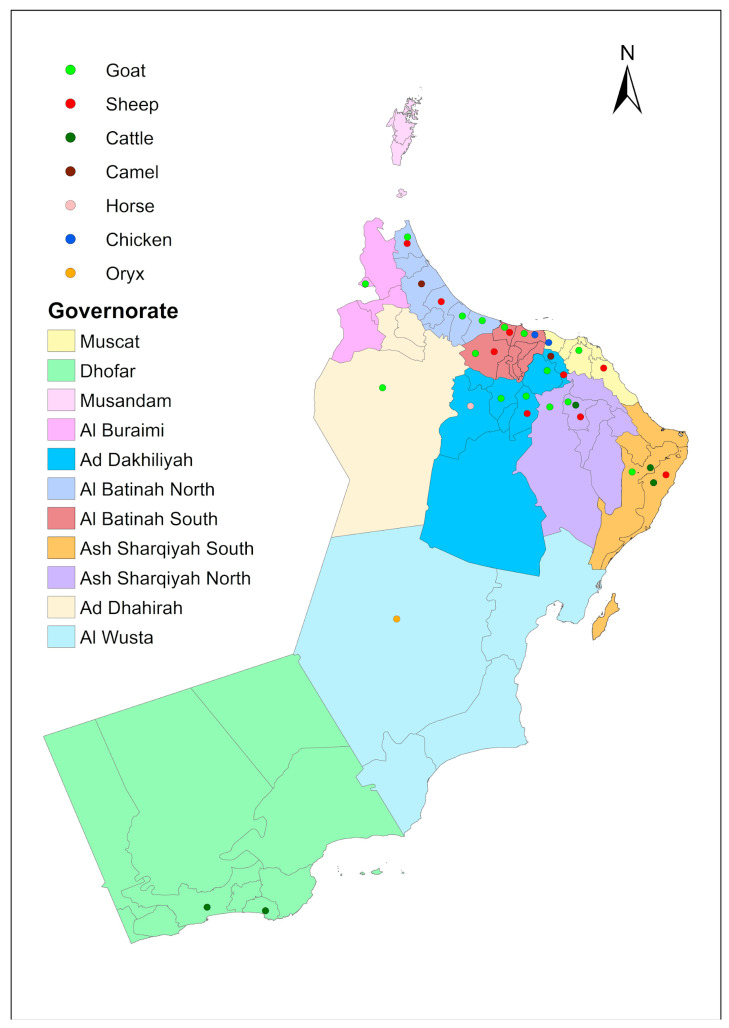
Geographical distribution of sampling for deceased animals. The map depicts the regions within the Sultanate of Oman where samples for deceased animals were collected.

A total of 295 consecutive, non-duplicate *E. coli* were included in the study, of which 104 were isolated from a total of 656 isolates obtained from clinical samples from urinary tract, bloodstream, and respiratory tract infections, 123 from 259 isolates obtained from diseased animals (goats, sheep, cattle, camel, oryx and poultry) across different governorates in the Sultanate of Oman (Figure 1), 50 from 105 fecal isolates from healthy animals, 14 from 35 isolates from sewage effluent, and 4 from 40 isolates from afalaj and wells (irrigation systems). The sampling area for human isolates was Sultan Qaboos University Hospital, Muscat, a quaternary referral hospital which caters to patients from across Oman. For deceased animals, the isolates were obtained from all the 11 governorates in Oman. The distribution of deceased animals is provided in Figure 5. Fecal samples from healthy animals were collected from Al Batinah South. Water samples were collected from wells and afalaj from the Al Dakhliya region. Sewage samples were collected from the Muscat region and Al Batinah South. The breakup of samples received from deceased animals were as follows: biopsies (21.1%, 26/123); feces (17.9%, 22/123); rectal swabs (16.3%, 20/123); urine (14.6%, 18/123); milk (11.4%, 14/123); nasal swabs (10.6%, 13/123); blood (4%, 5/123). The majority of the isolates were obtained from goats (34.1%, 42/123), followed by sheep (21.1%, 26/123). Fecal samples were collected from healthy animals. All samples were collected, transported and processed according to standard protocols [57,58,59,60].

Bacterial identification was performed using MALDI-TOF MS (Bruker, Munich, Germany) for clinical isolates at the diagnostic clinical laboratory at Sultan Qaboos University Hospital and at the Central Analytical and Applied Research Unit (CAARU) at Sultan Qaboos University’s College of Science for animal and environmental isolates. Only one isolate per patient or animal was included to avoid duplication. Antimicrobial susceptibility was performed by Phoenix^TM^ (BD Diagnostics, Franklin Lakes, NJ, USA) at SQUH for clinical isolates and by Kirby Bauer disk diffusion method for animal isolates [61]. The antimicrobials tested by disk diffusion were ampicillin (10 μg), amoxicillin-clavulanic acid (20/10 μg), piperacillin-tazobactam (10 μg), cefazolin, cefuroxime (30 µg), cefotaxime, ceftazidime (30 µg), ceftriaxone, cefepime (30 µg), and cefoxitin; carbapenems including imipenem (10 µg) and meropenem (10 µg); aminoglycosides including gentamicin (10 μg) and amikacin (30 μg); fluoroquinolones including ciprofloxacin (5 μg) and levofloxacin; trimethoprim-sulfamethoxazole; tetracycline and doxycycline; and nitrofurantoin (30 µg) (Oxoid, Basingstoke, UK).

ESBL was detected phenotypically in animal isolates by combined disk method, AmpC by disk approximation method in both human and animal isolates, and carbapenemase production by GeneXpert (Cepheid, Sunnyvale, CA, USA) in human isolates and lateral flow immunochromatographic assay (KPC/IMP/NDM/VIM/OXA-48 Combo Test Kit, Medomics, Nanjing, China) in animal isolates [62]. Sewage and water *E. coli* isolates were screened for ESBL and CRE carriage by ESBL and CRE CHROMagar (Paris, France). Confirmed isolates were stored at −20 °C in 50% glycerol until further analysis.

### 4.2. Whole Genome Sequencing and Bioinformatics Analysis

WGS was performed on a representative stratified subset of 50 isolates. The isolates were selected using a purposive stratified approach, whereby isolates were first grouped by source (human, animal, sewage) and resistance phenotype (ESBL vs. non-ESBL), and representative isolates were then intentionally selected from each group to enable cross-domain genomic comparison within available sequencing resources. The subset was designed to ensure representation across the major One Health interfaces and key phenotypic categories, with focus on ESBL-producing and non-ESBL comparator isolates. Accordingly, the WGS subset comprised human isolates (14 ESBLs and six non-ESBLs), animal isolates (10 ESBLs from deceased animals and 10 non-ESBLs from healthy animals), and sewage isolates (10 ESBLs). No water isolates were included in the WGS subset because no ESBL, AmpC, or carbapenem-resistant E. coli were identified in that compartment. This approach was intended to provide a representative cross-domain genomic comparison rather than a random or prevalence-estimating sample.

Sequencing was performed in the UK on the Illumina platform MicrobesNG, https://microbesng.com/, Birmingham, UK, accessed on 12 September 2024). Isolates were processed using commercial extraction kits according to the manufacturer’s protocol. DNA libraries were prepared following standard Illumina library preparation procedures and sequenced on an Illumina next-generation sequencing platform to generate paired-end reads. Raw sequence reads underwent quality control assessment, trimming, and de novo assembly. Assembled genomes were analyzed using various online tools from the Center for Genomic Epidemiology (CGE) (https://www.genomicepidemiology.org/, accessed on 18 November 2024) to identify multi-locus sequence types (MSLTs) [63], acquire antimicrobial resistance genes (ResFinder and the Comprehensive Antibiotic Resistance Database (CARD)), point mutations associated with antimicrobial resistance (ResFinder), plasmid replicons (PlasmidFinder), virulence factors (VirulenceFinder) and O:H serotypes (SerotypeFinder). A phylogenetic tree was constructed using CSI Phylogeny and the constructed tree was then visualized and annotated using Interactive Tree of Life (iTOL) [64] (accessed on 8 December 2024). Additionally, in silico Clermont phylotyper [65] (https://ezclermont.hutton.ac.uk/, accessed on 17 September 2025) was used for phylotype identification. All the genome sequences were submitted to NCBI, and accession numbers were awaited (submission ID: SUB16026812).

### 4.3. Statistical Analysis

Data was analyzed using IBM SPSS Statistics (Version 27). Categorical data were expressed as percentages. Comparative analyses were conducted to evaluate differences in resistance prevalence across human, animal, and environmental sources. Data were analyzed primarily using descriptive statistics. Categorical variables were summarized as counts and percentages, and comparative patterns across human, animal, and environmental sources were interpreted descriptively in view of the exploratory design of the study. All bar charts and pie charts were generated utilizing Microsoft Excel 2019. AI was employed to enhance a single graphic of the AMR heatmap.

## 5. Conclusions

This study highlights that *E. coli* across the human, animal, and environmental One Health interfaces in Oman exhibits limited direct genomic convergence. The human clinical interface emerged as the dominant reservoir of clinically relevant antimicrobial resistance, with the highest burden of ESBL (characterized predominantly by *bla*CTX-M15), AmpC, and carbapenem resistance, a broader resistome, and a richer virulome dominated by ExPEC-associated lineages and phylogroups. In contrast, animal and sewage isolates were genomically distinct in many respects, being more frequently associated with commensal or environmentally adapted phylogroups, and with *bla*CTX-M-55 linked resistance patterns. Although complete clonal overlap across the three domains was limited, shared resistance determinants, plasmid backbones, and selected sequence types were observed, particularly *bla*CTX-M-55 and IncF-family plasmids, supporting the possibility that gene flow across interfaces is mediated by mobile genetic elements. The findings suggest that Oman currently faces a pattern of parallel but connected AMR ecology, where clinically important resistance remains concentrated in the human healthcare sector, while animal and environmental compartments act as reservoirs of transmissible genetic platforms that could facilitate future convergence. Thus, continued integrated genomic surveillance across One Health interfaces is essential to detect emerging convergence events and to inform coordinated stewardship, infection control, veterinary policy, and environmental management strategies.

## 6. Limitations

This study should be interpreted in light of several limitations. It was conducted as a cross-sectional, exploratory One Health analysis, with non-duplicate *E. coli* isolates drawn from predefined human, animal, and environmental sample streams during the study period rather than a population-based national sampling frame. Thus, the findings are representative of the sampled study population and not direct prevalence estimates for Oman. In addition, environmental sampling was limited, particularly for water sources. No resistant water isolates were available for genomic comparison.

Furthermore, WGS was performed on a limited purposive, stratified subset of isolates to facilitate cross-domain comparison. The findings of this study are descriptive and hypothesis-generating, particularly given the modest size of some comparator groups. Larger longitudinal studies with broader sampling are recommended.

## Figures and Tables

**Table 1:** Distribution of different mutations conferring antimicrobial resistance among the isolates from different domains.

Domain	Isolate	Gene	Mutation	Antibiotics
Human	Ec_H06	*gyrA*	S83L	Ciprofloxacin, Nalidixic acid
	Ec_H08	*gyrA*	S83L, D87N	
		*parC*	S80I	
		*parE*	S458A	
	Ec_H09	*gyrA*	S83L, D87N	
		*parC*	S80I, E84V	
		*parE*	I529L	
	Ec_H12	*gyrA*	S83L, D87N	
		*parC*	S80I	
		*parE*	S458A	
	Ec_H15	*gyrA*	S83L, D87N	
		*parC*	S80I	
		*parE*	S458A	
	Ec_H16	*gyrA*	S83L, D87N	
		*parC*	S80I	
		*parE*	L416F	
	Ec_H18	*gyrA*	S83L, D87N	
		*parC*	S80I	
		*parE*	S458A	
	Ec_H20	*gyrA*	S83L	
Animal	Ec_A04	*gyrA*	S83L, D87N	Ciprofloxacin, Nalidixic acid
		*parC*	S80I, E84G	
	Ec_A06	*gyrA*	S83L, D87N	
		*parC*	S80I	
		*parE*	S458A	
	Ec_A07	*gyrA*	S83L, D87N	
		*parC*	S80I	
		*parE*	S458A	
	Ec_A10	*gyrA*	S83L, D87N	
		*parC*	S80I	
		*parE*	S458A	
	Ec_A11	*gyrA*	S83L, D87N	
		*parC*	S80I	
		*parE*	S458A	
	Ec_A13	*gyrA*	S83L, D87N	
		*parC*	S80I	
		*parE*	S458A	
	Ec_A14	*gyrA*	S83L, D87N	
		*parC*	S80I	
		*parE*	S458A	
	Ec_A16	*pmrB*	V161G	Colistin
	Ec_A17	*gyrA*	S83L, D87N	Ciprofloxacin, Nalidixic acid
		*parC*	S80I	
		*parE*	S458A	
	Ec_A19	*gyrA*	S83L, D87N	
		*parC*	S80I	
		*parE*	S458A	
	Ec_A20	*gyrA*	S83L, D87N	
		*parC*	S80I	
		*parE*	S458A	
Sewage	Ec_S01	*gyrA*	S83L, D87N	Ciprofloxacin, Nalidixic acid
		*parC*	S80I	
		*parE*	S458A	
	Ec_S02	*gyrA*	S83L, D87N	
		*parC*	S80I	
		*parE*	S458A	
	Ec_S03	*gyrA*	S83L, D87N	
		*parC*	S80I	
		*parE*	S458A	
	Ec_S04	*gyrA*	S83L, D87N	
		*parC*	S80I	
		*parE*	S458A	
	Ec_S05	*gyrA*	S83L, D87N	
		*parC*	S80I	
		*parE*	S458A	
	Ec_S06	*gyrA*	S83L, D87N	
		*parC*	S80I	
		*parE*	S458A	
	Ec_S07	*gyrA*	S83L, D87N	
		*parC*	S80I	
	Ec_S08	*gyrA*	S83L, D87N	
		*parC*	S80I	
	Ec_S09	*gyrA*	S83L, D87N	
		*parC*	S80I, E84G	
	Ec_S10	*gyrA*	S83L, D87N	
		*parC*	S80I	
		*parE*	S458A	

**Table 2:** MLST distribution of ESBL and non-ESBL *E. coli* across different domains of One Health.

MLST of Human-Derived *E. coli* Strains		MLST of Animal-Derived *E. coli* Strains		MSLT of Sewage-Derived *E. coli* Strains
Non-ESBL	ESBL	Non-ESBL	ESBL	ESBL
ST-73	ST-10	ST-101	ST-206	ST-155
ST-73	ST-10	ST-101	ST-224	ST-155
ST-421	ST-38	ST-155	ST-224	ST-156
ST-421	ST-46	ST-1139	ST-224	ST-167
ST-1485	ST-46	ST-1308	ST-224	ST-167
ST-1485	ST-73	ST-1485	ST-224	ST-167
	ST-127	ST-1952	ST-1196	ST-167
	ST-127	ST-2178	ST-1196	ST-167
	ST-131	ST-2522	ST-1196	ST-4985
	ST-224	ST-2741	ST-15570	ST-4985
	ST-361			
	ST-457			
	ST-924			
	ST-7401			

**Table 3:** Serotyping profile of isolates among the three domains based on O and H antigens.

Domain	Isolates	Serotyping		Domain	Isolates	Serotyping	
		H	O			H	O
**Human**	*Ec_H*01	H1	O6	**Animal**	*Ec_A*01	H31	O4
	*Ec_H*02	H42	O83		*Ec_A*02	H42	O83
	*Ec_H*03	H6	O11		*Ec_A*03	H25	O136
	*Ec_H*04	H7	O1		*Ec_A*04	H5	Unknown
	*Ec_H*05	H7	O1		*Ec_A*05	H40	O153
	*Ec_H*06	H18	O15		*Ec_A*06	H23	O78
	*Ec_H*07	Unknown	O6		*Ec_A*07	H10	Unknown
	*Ec_H*08	H30	O9, O9a		*Ec_A*08	H8	O185
	*Ec_H*09	H4	O25		*Ec_A*09	H12	O23
	*Ec_H*10	H1	O6		*Ec_A*10	H10	Unknown
	*Ec_H*11	H9	O9, O9a		*Ec_A*11	H23	O78
	*Ec_H*12	H30	O9a		*Ec_A*12	H4	O9
	*Ec_H*13	H21	O55		*Ec_A*13	H23	O78
	*Ec_H*14	H42	O83		*Ec_A*14	H23	O78
	*Ec_H*15	H10	O9a/O9		*Ec_A*15	H49	Unknown
	*Ec_H*16	H9	O101		*Ec_A*16	H28	Unknown
	*Ec_H*17	Unknown	O6		*Ec_A*17	H10	Unknown
	*Ec_H*18	H10	O9a/O9		*Ec_A*18	H40	O153
	*Ec_H*19	H18	O86		*Ec_A*19	H10	O109
	*Ec_H*20	H1	O6		*Ec_A*20	H23	O78
**Sewage**	*Ec_S*01	H23	O159	**Sewage**	*Ec_S*06	H10	O101
	*Ec_S*02	H10	O101		*Ec_S*07	H51	Unknown
	*Ec_S*03	H10	O101		*Ec_S*08	H51	Unknown
	*Ec_S*04	H10	O101		*Ec_S*09	H28	O54
	*Ec_S*05	H10	O101		*Ec_S*10	H23	O159

**Table 4:** Distribution of virulence genes in ESBL-producing *Escherichia coli* across One Health domains.

Virulence Gene	Function	Human n/14, %	Animal n/10, %	Sewage n/10, %	Total n/34, %	NCBI Description
Adhesion/Colonization Genes						
*afaA*	Adhesion/ Colonization	1/14, 7.14%	-	-	1/34, 2.94%	AfaVIII adhesin; member of afa-8 gene cluster; putative transcriptional regulator of the afa-8 gene cluster papI-papB family
*air*	Adhesion/ Colonization	1/14, 7.14%	-	-	1/34, 2.94%	
*csgA*	Adhesion/ Colonization	2/14, 14.29%	1/10, 10%	4/10 40%	7/34, 20.60%	Major subunit of curlin; it is actively secreted to the extracellular milieu, where CsgA monomers self-assemble into curli
*fimH*	Adhesion/ Colonization	8/14 57.14%	10/10 100%	10/10 100%	28/34, 82.35%	Type 1 fimbriae D-mannose specific adhesin
*focG*	Adhesion/ Colonization	1/14, 7.14%	-	-	1/34, 2.94%	F1C minor fimbrial subunit protein G precursor
*focIS*	Adhesion/ Colonization	2/14, 14.29%	-	-	2/34, 5.88%	
*iha*	Adhesion/ Colonization	1/14, 7.14%	-	-	1/34, 2.94%	Adhesin Iha adhesin
*ipfA*	Adhesion/ Colonization	-	5/10, 50%	5/10, 50%	10/34, 29.41%	Major fimbrial subunit IpfA, encodes a component of long polar fimbriae in diarrheagenic and extraintestinal pathogenic E. coli ExPEC, straini
*papC*	Adhesion/ Colonization	14/4, 28.57%	-	1/10, 10%	5/34, 14.71%	Outer membrane usher P fimbriae
*sfaD*	Adhesion/ Colonization	3/14, 21.43%	-	-	3/34, 8.82%	S fimbrial/F1C minor subunit
*sfaE*	Adhesion/ Colonization	2/14, 14.29%	-	-	2/34, 5.88%	S fimbrial/F1C minor subunit
*sfaS*	Adhesion/ Colonization	2/14, 14.29%	-	-	2/34, 5.88%	Sialic acid-binding adhesion
*tia*	Adhesion/ Colonization	-	-	1/10, 10%	1/34, 2.94%	Tia invasion determinant
*yehC*	Adhesion/ Colonization	-	-	2/10, 20%	2/34, 5.88%	Putative fimbrial chaperone
*yehD*	Adhesion/ Colonization	-	-	2/10, 20%	2/34, 5.88%	Fimbrial-like adhesin protein
Total adhesion/ colonization genes		11(27)	3(16)	7(25)	68	
Bacteriocin genes						
*cea*	Bacteriocins	-	5/10, 50%	1/10, 10%	6/34, 17.65%	Pore-forming bacteriocin colicin E1, encodes for Colicin E7 and Dr adhesins bind to CEA
*cia*	Bacteriocins	-	-	9/10, 90%	9/34, 26.47%	Colicin Ia protein
*cib*	Bacteriocins	-	1/10, 10%	4/10, 40%	5/34, 14.71%	Colicin ib/bacteriocin
*cma*	Bacteriocins	1/14, 7.14%	5/10, 50%	6/10, 60%	12/34, 35.29%	Colicin M activity protein
*colE*8	Bacteriocins	-	3/10, 30%	-	3/34, 8.82%	Colicin E8 DNase
*cvaC*	Bacteriocins	-	5/10, 50%	8/10, 80%	13/34, 38.24%	Microcin-V bacteriocin
*mchB*	Bacteriocins	1/14, 7.14%	-	-	1/34, 2.94%	Microcin H47/bactericidal antibiotic
*mchC*	Bacteriocins	1/14, 7.14%	-	-	1/34, 2.94%	MchC protein
*mchF*	Bacteriocins	1/14, 7.14%	-	-	1/34, 2.94%	ABC type transporter activity/ATP binding and hydrolysis/bacteriocin transport
Total bacteriocin genes		4 (4)	5 (19)	5 (28)	51	
Immune evasion/survival genes						
*Gad A*	Immune Evasion/Survival	14/10, 71.43%	-	-	10/34, 29.41%	
*Iss*	Immune Evasion/Survival	14/6, 42.85%	-	-	6/34, 17.65%	Increase serum survival lipoprotein Iss/resists the host’s complement system, sepsis
*KpsE*	Immune Evasion/Survival	14/5, 35.71%	-	-	5/34, 14.71%	Capsule polysaccharide export inner membrane protein/involved in the translocation of the polysialic acid capsule
*kpsMII*	Immune Evasion/Survival	14/5, 35.71%	-	-	5/34, 14.71%	Capsular polysaccharide synthesis K1
*ompT*	Immune Evasion/Survival	14/6, 42.85%	9/10, 90%	1/10, 10%	16/34, 47.06%	Outer membrane protease protein protease 7/degrades antimicrobial peptides
*Pic*	Immune Evasion/Survival	1/14, 7.14%	-	-	1/34, 2.94%	Serine protease pic autotransporter
*traT*	Immune Evasion/Survival	14/5, 35.71%	10/10, 100%	8/10, 80%	23/34, 67.65%	Complement resistance protein precursor TraT/resists killing by the host’s immune system serum resistance by interfering with complement deposition and reducing phagocytosis
*terC*	Immune Evasion/Survival	14/3, 21.43%	4/10, 40%	7/10, 70%	14/34, 41.18%	Tellurium resistance membrane protein TerC
Total immune evasion/survival genes		8/8 (42)	3/8 (23)	3/8 (16)	66	
Iron acquisition genes						
*chuA*	Iron Acquisition	14/7, 50%	-	-	7/34, 20.59%	TonB-dependent heme/hemoglobin receptor
*fyuA*	Iron Acquisition	8/14, 57.14%	-	1/10, 10%	9/34, 26.47%	Ferric yersiniabactin uptake receptor FyuA
*IreA*	Iron Acquisition	1/14, 7.14%	-	-	1/34, 2.94%	TonB-dependent siderophore receptor IreA
*iroN*	Iron Acquisition	2/14, 14.29%	4/10, 40%	3/10, 30%	9/34, 26.47%	Enterobactin catecholate siderophore receptor protein/encodes receptor which scavenges iron in iron-poor environments
*irp*2	Iron Acquisition	14/3, 21.43%	-	-	-	High-molecular-weight protein 2 nonribosomal peptide synthetase
*iucC*	Iron Acquisition	2/14, 14.29%	10/10, 100%	2/10, 20%	14/34, 41.17%	Aerobactin synthetase
*iutA*	Iron Acquisition	1/14, 7.14%	6/10, 60%	3/10, 30%	10/34, 29.41%	Ferric aerobactin receptor
*sitA*	Iron Acquisition	14/10, 71.43%	10/10, 100%	8/10, 80%	28/34, 82.35%	Iron/manganese ABC transporter substrate-binding protein/transports ferrous iron and manganese
Total iron acquisition genes		8 (35)	4 (30)	4 (17)	82	
*aaiC*	Secretion/Regulation	1/14, 7.14%	-	-	-	Type VI secretion system protein AaiC/Hcp2
*capU*	Secretion/Regulation	2/14, 14.29%	-	-	-	Putative hexosyltransferase CapU
*eilA*	Secretion/Regulation	1/14, 7.14%	-	-	-	HilA-homolog/putative transcriptional regulator of ETT2 associated genes
*etsC*	Secretion/Regulation	-	-	1/10, 10%	-	Putative type I secretion outer membrane protein
*hha*	Secretion/Regulation	1/14, 7.14%	-	-	-	Hemolysin expression-modulating protein Hha
*traJ*	Secretion/Regulation	2/14, 14.29%	-	2/10, 20%	4/34, 11.70%	Transfer of plasmid RP4 during bacterial conjugation requiring the plasmid-encoded TraJ protein/relaxosome protein
Total secretion/regulation genes		5 (7)	-	2 (3)	10	
Toxins/genotoxin genes						
*astA*	Toxins/Genotoxins	-	5/10, 50%	7/10, 70%	-	pAA
*cibB*	Toxins/Genotoxins	2/14, 14.29%	-	-	2/34, 5.88%	Fratricide two-peptide bacteriocin subunit
*cnf*1	Toxins/Genotoxins	1/14, 7.14%	-	-	1/34, 2.94%	Cytotoxic necrotizing factor 1
*hlyA*	Toxins/Genotoxins	1/14, 7.14%	-	-	1/34, 2.94%	Hemolysin A
*hlyE*	Toxins/Genotoxins	1/14, 7.14%	-	4/10, 40%	5/34, 14.7%	Hemolysin E
*hlyF*	Toxins/Genotoxins	2/14, 14.29%	-	6/10, 60%	8/34, 23.53%	Cytoplasmic enzyme that increases the formation of outer membrane vesicles allowing the release of haemolysin E
*USP*	Toxins/Genotoxins	-	-	-	-	Uropathogenic-specific protein
Total toxins/genotoxin genes		5 (7)	1 (5)	3 (17)	29	
Total genes (total) isolates		41 (122)	16 (93)	24 (106)		
		Color scale.				
						10–39% prevalence
						40–59% prevalence
						60–79% prevalence
						80–100% prevalence

## Data Availability

The original contributions presented in this study are included in the article. Further inquiries can be directed to the corresponding author.

## Supplementary Materials

The following supporting information can be downloaded at: https://www.mdpi.com/article/10.3390/antibiotics15040411/s1, File S1 and Table S1: Distribution of *E. coli* across the three interfaces; Figure S1. Quanti-tray used for identification of *E. coli* and coliforms; Table S2: Prevalence of Antimicrobial Resistance Genes Across the three interfaces [57,58,59].

## Abbreviations

The following abbreviations are used in this manuscript:

AMR	Antimicrobial Resistance
ESBL	Extended-Spectrum Beta-Lactamase
Non-ESBL	Non-Extended-Spectrum Beta-Lactamase
MLST	Multi-Locus Sequence Typing
ExPEC	Extraintestinal Pathogenic *Escherichia coli*
WHO	World Health Organization
WGS	Whole Genome Sequencing

## References

[1] McEwen S.A., Collignon P.J. (2018). Antimicrobial Resistance: A One Health Perspective. Microbiol. Spectr..

[2] Aslam B., Khurshid M., Arshad M.I., Muzammil S., Rasool M., Yasmeen N., Shah T., Chaudhry T.H., Rasool M.H., Shahid A. (2021). Antibiotic Resistance: One Health One World Outlook. Front. Cell. Infect. Microbiol..

[3] WHO (2021). WHO Integrated Global Surveillance on ESBL-Producing E. coli Using a “One Health” Approach: Implementation and Opportunities.

[4] Leverstein-van Hall M.A., Dierikx C.M., Cohen Stuart J., Voets G.M., van den Munckhof M.P., van Essen-Zandbergen A., Platteel T., Fluit A.C., van de Sande-Bruinsma N., Scharinga J. (2011). Dutch Patients, Retail Chicken Meat and Poultry Share the Same ESBL Genes, Plasmids and Strains. Clin. Microbiol. Infect..

[5] Kluytmans J.A.J.W., Overdevest I.T.M.A., Willemsen I., Kluytmans-Van Den Bergh M.F.Q., Van Der Zwaluw K., Heck M., Rijnsburger M., Vandenbroucke-Grauls C.M.J.E., Savelkoul P.H.M., Johnston B.D. (2013). Extended-Spectrum β-Lactamase–Producing *Escherichia coli* From Retail Chicken Meat and Humans: Comparison of Strains, Plasmids, Resistance Genes, and Virulence Factors. Clin. Infect. Dis..

[6] Ludden C., Raven K.E., Jamrozy D., Gouliouris T., Blane B., Coll F., de Goffau M., Naydenova P., Horner C., Hernandez-Garcia J. (2019). One Health Genomic Surveillance of *Escherichia coli* Demonstrates Distinct Lineages and Mobile Genetic Elements in Isolates from Humans versus Livestock. mBio.

[7] Siscar-Lewin S., Hube B., Brunke S. (2022). Emergence and Evolution of Virulence in Human Pathogenic Fungi. Trends Microbiol..

[8] Jiang J., Wang L., Hu Y., Chen X., Li P., Zhang J., Zhang Y., Su J., Xu X., Xiao Y. (2025). Global Emergence of Carbapenem-Resistant Hypervirulent *Klebsiella pneumoniae* Driven by an IncFIIK34 KPC-2 Plasmid. eBioMedicine.

[9] Biggel M., Moons P., Nguyen M.N., Goossens H., Van Puyvelde S. (2022). Convergence of Virulence and Antimicrobial Resistance in Increasingly Prevalent *Escherichia coli* ST131 PapGII+ Sublineages. Commun. Biol..

[10] Mazumder R., Abdullah A., Ahmed D., Hussain A. (2020). High Prevalence of *bla*CTX-M-15 Gene among Extended-Spectrum β-Lactamase-Producing *Escherichia coli* Isolates Causing Extraintestinal Infections in Bangladesh. Antibiotics.

[11] Zurita J., Sevillano G., y Miño A.P., Haro N., Larrea-Álvarez M., Alcocer I., Ortega-Paredes D. (2023). Dominance of ST131, B2, *bl*aCTX-M-15, and PapA-PapC-KpsMII-UitA among ESBL *Escherichia coli* Isolated from Bloodstream Infections in Quito, Ecuador: A 10-Year Surveillance Study (2009–2019). J. Appl. Microbiol..

[12] Kim J.I., Moon B.Y., Ali M.S., Kang H.S., Choi J.H., Kim J.M., Park S.C., Lim S.K. (2025). High Prevalence of *bla*CTX-M-55-Carrying *Escherichia coli* in Both Ceftiofur-Use and Non-Use Pig Farms. Appl. Environ. Microbiol..

[13] Yu K., Huang Z., Xiao Y., Bai X., Gao H., Wang D. (2024). Epidemiology and Molecular Characterization of CTX-M-Type ESBLs Producing *Escherichia coli* Isolated from Clinical Settings. J. Glob. Antimicrob. Resist..

[14] Sellera F.P., Fuentes-Castillo D., Furlan J.P.R. (2023). One Health Spread of 16S Ribosomal RNA Methyltransferase-Harboring Gram-Negative Bacterial Genomes: An Overview of the Americas. Pathogens.

[15] Zhang H., Luo Y., Zhu X., Ju F., Zhang H., Luo Y., Zhu X., Ju F. (2025). Environmental Antimicrobial Resistance Key Reservoirs, Surveillance and Mitigation under One Health. Biocontaminant.

[16] Banjo O.A., Adekanmbi A.O., Akinbola O.J., Thomas B.T., Ilusanya O.A.F. (2025). Molecular Characterization of Plasmid-Mediated Quinolone Resistance Genes in Multidrug-Resistant *Escherichia coli* Isolated From Wastewater Generated From the Hospital Environment. Environ. Health Insights.

[17] Ajulo S., Awosile B. (2024). Global Antimicrobial Resistance and Use Surveillance System (GLASS 2022): Investigating the Relationship between Antimicrobial Resistance and Antimicrobial Consumption Data across the Participating Countries. PLoS ONE.

[18] Venkateswaran P., Vasudevan S., David H., Shaktivel A., Shanmugam K., Neelakantan P., Solomon A.P. (2023). Revisiting ESKAPE Pathogens: Virulence, Resistance, and Combating Strategies Focusing on Quorum Sensing. Front. Cell. Infect. Microbiol..

[19] Domínguez M., Miranda C.D., Fuentes O., De La Fuente M., Godoy F.A., Bello-Toledo H., González-Rocha G. (2019). Occurrence of Transferable Integrons and Suland Dfrgenes among Sulfonamide-and/or Trimethoprim-Resistant Bacteria Isolated from Chilean Salmonid Farms. Front. Microbiol..

[20] Zalewska M., Błażejewska A., Czapko A., Popowska M. (2021). Antibiotics and Antibiotic Resistance Genes in Animal Manure—Consequences of Its Application in Agriculture. Front. Microbiol..

[21] Chekole W.S., Potgieter L., Adamu H., Sternberg-Lewerin S., Tessema T.S., Magnusson U. (2025). Genomic Insights into Antimicrobial Resistance and Virulence of E. Coli in Central Ethiopia: A One Health Approach. Front. Microbiol..

[22] Aworh M.K., Kwaga J.K.P., Hendriksen R.S., Okolocha E.C., Harrell E., Thakur S. (2023). Quinolone-Resistant *Escherichia coli* at the Interface between Humans, Poultry and Their Shared Environment- a Potential Public Health Risk. One Health Outlook.

[23] Maurya N., Jangra M., Tambat R., Nandanwar H. (2019). Alliance of Efflux Pumps with β-Lactamases in Multidrug-Resistant *Klebsiella pneumoniae* Isolates. Microb. Drug Resist..

[24] Kumar H., Chen B.H., Kuca K., Nepovimova E., Kaushal A., Nagraik R., Bhatia S.K., Dhanjal D.S., Kumar V., Kumar A. (2020). Understanding of Colistin Usage in Food Animals and Available Detection Techniques: A Review. Animals.

[25] Lin J.C., Kristopher Siu L.K., Chang F.Y., Wang C.H. (2024). Mutations in the PmrB Gene Constitute the Major Mechanism Underlying Chromosomally Encoded Colistin Resistance in Clinical *Escherichia coli*. J. Glob. Antimicrob. Resist..

[26] Manges A.R., Geum H.M., Guo A., Edens T.J., Fibke C.D., Pitout J.D.D. (2019). Global Extraintestinal Pathogenic *Escherichia coli* (Expec) Lineages. Clin. Microbiol. Rev..

[27] Riley L.W. (2020). Distinguishing Pathovars from Nonpathovars: *Escherichia coli*. Microbiol. Spectr..

[28] Dos Anjos Adur M., Châtre P., Métayer V., Drapeau A., Pillonetto M., Penkal M.L., Lopes J.K., Beirão B.C.B., Madec J.Y., de Macedo R.E.F. (2022). *Escherichia coli* ST224 and IncF/BlaCTX-M-55 Plasmids Drive Resistance to Extended-Spectrum Cephalosporins in Poultry Flocks in Parana, Brazil. Int. J. Food Microbiol..

[29] Benlabidi S., Raddaoui A., Lengliz S., Cheriet S., Hynds P., Achour W., Ghrairi T., Abbassi M.S. (2023). Occurrence of High-Risk Clonal Lineages ST58, ST69, ST224, and ST410 among Extended-Spectrum β-Lactamase-Producing *Escherichia coli* Isolated from Healthy Free-Range Chickens (*Gallus gallus domesticus*) in a Rural Region in Tunisia. Genes.

[30] Mujahid F., Rasool M.H., Shafiq M., Aslam B., Khurshid M. (2024). Emergence of Carbapenem-Resistant Uropathogenic *Escherichia coli* (ST405 and ST167) Strains Carrying *bla*CTX-M-15, *bla*NDM-5 and Diverse Virulence Factors in Hospitalized Patients. Pathogens.

[31] Gomi R., Matsumura Y., Yamamoto M., Tanaka M., Komakech A.J., Matsuda T., Harada H. (2024). Genomic Surveillance of Antimicrobial-Resistant *Escherichia coli* in Fecal Sludge and Sewage in Uganda. Water Res..

[32] Huang J., Zhu J., Gong D., Wu L., Zhu Y., Hu L. (2022). Whole Genome Sequence of EC16, a *bla*NDM-5-, *bla*CTX-M-55-, and *fosA3*-Coproducing *Escherichia coli* ST167 Clinical Isolate from China. J. Glob. Antimicrob. Resist..

[33] Kawano K., Masaki T., Kawaguchi T., Kuroda M. (2025). Persistence of Colistin Resistance and *mcr-1.1*-Positive *E. Coli* in Poultry Despite Colistin Ban in Japan. Antibiotics.

[34] Gilrane V.L., Lobo S., Huang W., Zhuge J., Yin C., Chen D., Alvarez K.J., Budhai A., Nadelman I., Dimitrova N. (2017). Complete Genome Sequence of a Colistin-Resistant *Escherichia coli* Strain Harboring *mcr-1* on an IncHI2 Plasmid in the United States. Genome Announc..

[35] Wardoyo E.H., Sugawara Y., Nakano S., Zuo H., Elahi S., Arai C., Kondo K., Kuntaman K., Sugai M. (2025). Genomic Characterization of Extended-Spectrum β-Lactamase-Producing *Escherichia coli* Spread among Chickens and Healthy Residents in Lombok, Indonesia. Appl. Environ. Microbiol..

[36] Müller A., Stephan R., Nüesch-Inderbinen M. (2016). Distribution of Virulence Factors in ESBL-Producing *Escherichia coli* Isolated from the Environment, Livestock, Food and Humans. Sci. Total Environ..

[37] Royer G., Darty M.M., Clermont O., Condamine B., Laouenan C., Decousser J.W., Vallenet D., Lefort A., de Lastours V., Denamur E. (2021). Phylogroup Stability Contrasts with High within Sequence Type Complex Dynamics of *Escherichia coli* Bloodstream Infection Isolates over a 12-Year Period. Genome Med..

[38] Coura F.M., de Araújo Diniz S., Mussi J.M.S., Silva M.X., Lage A.P., Heinemann M.B. (2017). Characterization of Virulence Factors and Phylogenetic Group Determination of *Escherichia coli* Isolated from Diarrheic and Non-Diarrheic Calves from Brazil. Folia Microbiol..

[39] Watt A.E., Cummins M.L., Donato C.M., Wirth W., Porter A.F., Andersson P., Donner E., Huynh T., Phabmixay A., Flynn E. (2025). Parameters for One Health Genomic Surveillance of *Escherichia coli* from Australia. Nat. Commun..

[40] Zakaria A.S., Edward E.A., Mohamed N.M. (2022). Pathogenicity Islands in Uropathogenic *Escherichia coli* Clinical Isolate of the Globally Disseminated O25:H4-ST131 Pandemic Clonal Lineage: First Report from Egypt. Antibiotics.

[41] Peirano G., Pitout J.D.D. (2010). Molecular Epidemiology of *Escherichia coli* Producing CTX-M Beta-Lactamases: The Worldwide Emergence of Clone ST131 O25:H4. Int. J. Antimicrob. Agents.

[42] Yumuk Z., Afacan G., Nicolas-Chanoine M.H., Sotto A., Lavigne J.P. (2008). Turkey: A Further Country Concerned by Community-Acquired *Escherichia coli* Clone O25-ST131 Producing CTX-M-15. J. Antimicrob. Chemother..

[43] Guo Y., Xiao R., Feng J., Wang X., Lai J., Kang W., Li Y., Zhu X., Ji T., Huang X. (2024). Distribution of Virulence Genes and Antimicrobial Resistance of *Escherichia coli* Isolated from Hospitalized Neonates: A Multi-Center Study across China. Heliyon.

[44] James E.M., Kimera Z.I., Mgaya F.X., Niccodem E.M., Efraim J.E., Matee M.I., Mbugi E.V. (2025). Occurrence of Virulence Genes in Multidrug-Resistant *Escherichia coli* Isolates from Humans, Animals, and the Environment: One Health Perspective. PLoS ONE.

[45] Abeni B.A., Frank-Peterside N., Otokunefor K. (2024). Comparative Analysis of Virulence Gene Profiles of *Escherichia coli* from Human and Non-Human Sources in Rivers State, Nigeria. Access Microbiol..

[46] Sora V.M., Meroni G., Martino P.A., Soggiu A., Bonizzi L., Zecconi A. (2021). Extraintestinal Pathogenic *Escherichia coli*: Virulence Factors and Antibiotic Resistance. Pathogens.

[47] Gagaletsios L.A., Kikidou E., Galbenis C., Bitar I., Papagiannitsis C.C. (2025). Exploring Virulence Characteristics of Clinical *Escherichia coli* Isolates from Greece. Microorganisms.

[48] Rizzo L., Manaia C., Merlin C., Schwartz T., Dagot C., Ploy M.C., Michael I., Fatta-Kassinos D. (2013). Urban Wastewater Treatment Plants as Hotspots for Antibiotic Resistant Bacteria and Genes Spread into the Environment: A Review. Sci. Total Environ..

[49] Manaia C.M., Rocha J., Scaccia N., Marano R., Radu E., Biancullo F., Cerqueira F., Fortunato G., Iakovides I.C., Zammit I. (2018). Antibiotic Resistance in Wastewater Treatment Plants: Tackling the Black Box. Environ. Int..

[50] Del Carmen Rocha-Gracia R., Lozano-Zarain P., Cázarez Z.G., Alonso C.A., Brambila E., Torres C., Cortés-Cortés G. (2022). IncFIB Plasmids Carrying the Resistance Gene *bla*CTX-M-15 in ESBL-Producing *Escherichia coli* Clones from Pediatric Patients. J. Infect. Dev. Ctries..

[51] Wang H., Xu Q., Chen K., Chan B.K.W., Ye L., Yang X., Xie M., Liu X., Ni H., Chan E.W.C. (2022). A Siderophore-Encoding Plasmid Encodes High-Level Virulence in *Escherichia coli*. Microbiol. Spectr..

[52] Hoshiko Y., Chowdhury G., Kitahara K., Ghosh D., Nagano D.S., Ohno A., Miyoshi S.I., Okuno M., Yamamoto T., Dutta S. (2025). Genomic Features of Three Major Diarrhoeagenic *Escherichia coli* Pathotypes in India. Microb. Genom..

[53] Khajanchi B., Felix M., Sopovski D., Yoskiwotiz N., Abott C., Grim C., Foley S. (2021). Comparative Analyses and Virulence Potential of Incompatibility Group FIB Plasmid Containing Salmonella Schwarzengrund Strains Isolated from Food and Clinical Sources.

[54] Rodríguez-Navarro J., Miró E., Brown-Jaque M., Hurtado J.C., Moreno A., Muniesa M., González-López J.J., Vila J., Espinal P., Navarro F. (2020). Comparison of Commensal and Clinical Isolates for Diversity of Plasmids in *Escherichia coli* and *Klebsiella pneumoniae*. Antimicrob. Agents Chemother..

[55] Salinas L., Cárdenas P., Johnson T.J., Vasco K., Graham J., Trueba G. (2019). Diverse Commensal *Escherichia coli* Clones and Plasmids Disseminate Antimicrobial Resistance Genes in Domestic Animals and Children in a Semirural Community in Ecuador. mSphere.

[56] Ramatla T., Mafokwane T., Lekota K., Monyama M., Khasapane G., Serage N., Nkhebenyane J., Bezuidenhout C., Thekisoe O. (2023). “One Health” Perspective on Prevalence of Co-Existing Extended-Spectrum β-Lactamase (ESBL)-Producing *Escherichia coli* and *Klebsiella pneumoniae*: A Comprehensive Systematic Review and Meta-Analysis. Ann. Clin. Microbiol. Antimicrob..

[57] Dean Z.S., Stott K., Schubert W., Seto E.P., Chandrapati S. (2023). Dehydrated Thin Film Media to Rapidly Estimate Bioburden for Planetary Protection Flight Implementation. Int. J. Astrobiol..

[58] Saxena S., Chakraborty D. (2023). Identification and Characterization of Water Borne Pathogen. South Asian J. Res. Microbiol..

[59] Choudhary A.K., Fong K., Grainger E., Kumar R., Melanson R., Salisbury D., Carrera F., Nikitina S., Halder A., Thakur R. (2017). Improving Water Quality in the Villages of Himachal Pradesh.

[60] (2022). Analysis and Presentation of Cumulative Antimicrobial Susceptibility Test Data.

[61] (2024). Performance Standards for Antimicrobial Susceptibility Testing, 32nd Ed.

[62] AL Shizawi N., AL Jabri Z., Khan F., Sami H., AL Siyabi T., AL Muharrmi Z., Sirasanagandla S.R., Rizvi M. (2025). Mapping Antimicrobial Resistance in *Escherichia coli* and *Klebsiella pneumoniae* from Complicated Urinary Tract Infections in Oman: Phenotypic and Genotypic Insights. Diagnostics.

[63] Larsen M.V., Cosentino S., Rasmussen S., Friis C., Hasman H., Marvig R.L., Jelsbak L., Sicheritz-Pontén T., Ussery D.W., Aarestrup F.M. (2012). Multilocus Sequence Typing of Total-Genome-Sequenced Bacteria. J. Clin. Microbiol..

[64] Letunic I., Bork P. (2024). Interactive Tree of Life (ITOL) v6: Recent Updates to the Phylogenetic Tree Display and Annotation Tool. Nucleic Acids Res..

[65] Waters N.R., Abram F., Brennan F., Holmes A., Pritchard L. (2020). Easy Phylotyping of *Escherichia coli* via the EzClermont Web App and Command-Line Tool. Access Microbiol..

