# Supplementary material for: Genomic Epidemiology of ESBL and Non-ESBL-Producing Escherichia coli Across One Health Interfaces in Oman
Source: Antibiotics (Basel). 2026 Apr 17;15(4):411. doi: 10.3390/antibiotics15040411 (PMC13114006; doi:10.3390/antibiotics15040411)
Supplement: Supplementary file 1 [file antibiotics-15-00411-s001.zip › Supplementary File S1_Distribution of E coli across the three interfaces.pdf]

Supplementary document S1

Collection and Processing of sewage samples:

Raw untreated sewage samples were collected from agricultural farms in the Muscat and Al-Batinah regions over a three-month period from June 2024 to August 2024. One-liter samples were placed into sterile bottles, transported to the Central Laboratory of Animal Health using ice packs in portable insulated containers, and processed within 24 hours. Samples were concentrated through the filtration technique utilizing 0.2-μm pore size filter membranes as described by Dean et al. Membranes were applied to the surface of selective chromogenic media- ESBL CHROMagar and CRE CHROMagar. These plates were incubated for 24 hours at 37 °C in the incubator and read after 24 hours [1].

Drinking water samples:

Samples were collected from wells and falaj systems using Colilert for the detection of Total Coliforms and *E. coli*. village [2]. The procedure involved the combination of 100 ml samples with one snap pack of Colilert in a sterile container. The mixture was subsequently added to a Quanti-tray and incubated at 35°C ± 0.5°C for 24 hours. The detection of total coliforms and *E. coli* was conducted by directly examining the incubated Quanti-tray under UV light [3]. Yellow wells signified the presence of coliforms, whereas wells that exhibited both yellow and fluorescent characteristics indicated the presence of *E. coli*, as illustrated in figure 1 below. Following the receipt of samples from NAMA company, the yellow or positive wells underwent additional testing in the microbiology laboratory at the Central Laboratory of Animal Health. The initial step involved culturing on MacConkey agar. Subsequently, further identification was conducted using MALDI BIOTYPER at the Central Analytical and Applied Research Unit (CAARU). Positive *E.coli* positive samples were then inoculated onto ESBL CHROMagar and CRE CHROMagar for screening of ESBL and CRE.

Figure S1. Quanti-tray used for identification of *E. coli* and coliforms.

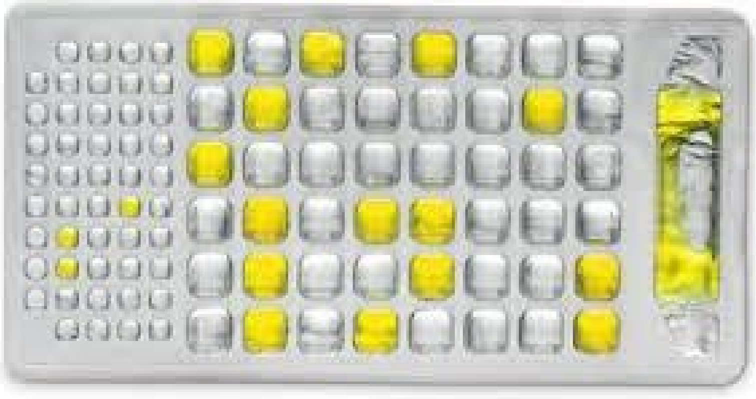

References

1. Dean, Z.S.; Stott, K.; Schubert, W.; Seto, E.P.; Chandrapati, S. Dehydrated Thin Film Media to Rapidly Estimate Bioburden for Planetary Protection Flight Implementation. *Int. J. Astrobiol.* **2023**, *22*, 568–582, doi:10.1017/S1473550423000149.
2. Saxena S, C.D. Identification and Characterization of Water Borne Pathogen. *South Asian Journal of Research in Microbiology* **2023**, *17*, 7–12.
3. Choudhary, A.K.; Fong, K.; Grainger, E.; Kumar, R.; Melanson, R.; Salisbury, D.; Carrera, F.; Nikitina, S.; Halder, A.; Thakur, R. Improving Water Quality in the Villages of Himachal Pradesh. **2017**.
