# Supplementary material for: Genomic Epidemiology of ESBL and Non-ESBL-Producing Escherichia coli Across One Health Interfaces in Oman
Source: Antibiotics (Basel). 2026 Apr 17;15(4):411. doi: 10.3390/antibiotics15040411 (PMC13114006; doi:10.3390/antibiotics15040411)
Supplement: Supplementary file 1 [file antibiotics-15-00411-s001.zip › Supplementary Table S1_Distribution of E coli across the three interfaces.pdf]

Supplementary Table S1. Distribution of *E. coli* across the three Health interfaces

**Interface**	***E. coli* (n)**	**ESBL (%)**	**AmpC (%)**	**CRE (%)**
Humans	104	73.0	11.5	4.8
**Animals (Total)**	173			-
Diseased	123	16.3	6.5	-
Healthy	50	8	-	-
**Environment (Total)**	14	-	-	-
Drinking water from wells	4	-	-	-
Sewage	10	28.6%	-	-
