# Supplementary material for: Genomic Epidemiology of ESBL and Non-ESBL-Producing Escherichia coli Across One Health Interfaces in Oman
Source: Antibiotics (Basel). 2026 Apr 17;15(4):411. doi: 10.3390/antibiotics15040411 (PMC13114006; doi:10.3390/antibiotics15040411)
Supplement: Supplementary file 1 [file antibiotics-15-00411-s001.zip › Supplementary Table S2_Prevalence of Antimicrobial Resistance Genes Across the three interfaces.pdf]

Supplementary Table S2. Distribution of Antimicrobial Resistance Genes of *E. coli* across the three Health interfaces

AMR Gene	Mechanism of Resistance	Human ESBL *E. coli* (n=14)	Human Non-ESBL *E. coli* (n=6)	Animal ESBL E. coli (n=10)	Animal Non ESBL *E. coli* (n=10)	Sewage ESBL *E. coli* (n=10)
marA	Antibiotic efflux; reduced permeability to antibiotic	**14/14, 100%**	2/6	**10/10, 100%**	8/10, 80%	**10/10, 100%**
acrB	Antibiotic efflux	**14/14, 100%**	**6/6, 100%**	**10/10, 100%**	9/10, 90%	**10/10, 100%**
acrD	Antibiotic efflux	13/14, 92.9%		**10/10, 100%**		**10/10, 100%**
AcrE	Antibiotic efflux	**14/14, 100%**	**6/6, 100%**	**10/10, 100%**	9/10, 90%	**10/10, 100%**
AcrF	Antibiotic efflux	**14/14, 100%**		**10/10, 100%**		**10/10, 100%**
AcrS	Antibiotic efflux	**14/14, 100%**	2/6, 33/3%	**10/10, 100%**	4/10, 40%	**10/10, 100%**
Escherichia coli acrA	Antibiotic efflux	**14/14, 100%**	2/6, 33/3%	**10/10, 100%**	9/10, 90%	**10/10, 100%**
mdtA	Antibiotic efflux	**14/14, 100%**		**10/10, 100%**		**10/10, 100%**
mdtC	Antibiotic efflux	**14/14, 100%**		**10/10, 100%**		**10/10, 100%**
mdtB	Antibiotic efflux	**14/14, 100%**		**10/10, 100%**		**10/10, 100%**
mdtN	Antibiotic efflux	**14/14, 100%**		**10/10, 100%**	1/10, 10%	**10/10, 100%**
TolC	Antibiotic efflux	**14/14, 100%**		**10/10, 100%**	**10/10, 100%**	**10/10, 100%**
cpxA	Antibiotic efflux	13/14, 92.9%	4/6, 66.6%	9/10, 90%	8/10, 80%	9/10, 90%
mdtH	Antibiotic efflux	**14/14, 100%**		**10/10, 100%**		**10/10, 100%**
mdtE	Antibiotic efflux	**14/14, 100%**	2/6, 33/3%	**10/10, 100%**	8/10, 80%	**10/10, 100%**
mdtF	Antibiotic efflux	**14/14, 100%**		**10/10, 100%**	1/10, 10%	**10/10, 100%**
mdtG	Antibiotic efflux	**14/14, 100%**	2/6, 33/3%	**10/10, 100%**	5/10, 50%	**10/10, 100%**

mdtO	Antibiotic efflux	**14/14, 100%**		**10/10, 100%**		**10/10, 100%**
kdpF	Antibiotic efflux	9/14, 64.2%		4/10, 40%		7/10, 70%
kdpE	Antibiotic efflux	13/14, 92.9%		**10/10, 100%**		**10/10, 100%**
H-NS	Antibiotic efflux	**14/14, 100%**	**6/6, 100%**	**10/10, 100%**	9/10, 90%	**10/10, 100%**
emrA	Antibiotic efflux	**14/14, 100%**		**10/10, 100%**		**10/10, 100%**
emrB	Antibiotic efflux	**14/14, 100%**	2/6, 33/3%	**10/10, 100%**	6/10, 60%, 60%	**10/10, 100%**
emrK	Antibiotic efflux	**14/14, 100%**		**10/10, 100%**	3/10, 30%, 30%	**10/10, 100%**
emrR	Antibiotic efflux	**14/14, 100%**	4/6, 66.6%	**10/10, 100%**	4/10, 40%	**10/10, 100%**
emrY	Antibiotic efflux	**14/14, 100%**		**10/10, 100%**	3/10, 30%, 30%	**10/10, 100%**
mdtM	Antibiotic efflux	11/14, 78.5%		**10/10, 100%**		**10/10, 100%**
mdtP	Antibiotic efflux	**14/14, 100%**		**10/10, 100%**		**10/10, 100%**
evgA	Antibiotic efflux	**14/14, 100%**	**6/6, 100%**	**10/10, 100%**	7/10, 70%	**10/10, 100%**
evgS	Antibiotic efflux	13/14, 92.9%		**10/10, 100%**		**10/10, 100%**
msbA	Antibiotic efflux	12/14, 85.7%	2/6, 33/3%	9/10, 90%	7/10, 70%	8/10, 80%
gadX	Antibiotic efflux	**14/14, 100%**		4/10, 40%	2/10, 20%	**10/10, 100%**
leuO	Antibiotic efflux	12/14, 85.7%		**10/10, 100%**		9/10, 90%
qacL	Antibiotic efflux			1/10, 10%		4/10, 40%
qacEdelta1	Antibiotic efflux	3/14, 21.4%		0/10, 0%		6/10, 60%
CRP	Antibiotic efflux			**10/10, 100%**		9/10, 90%
gadW	Antibiotic efflux	4/14, 28.5%	4/6, 66.6%	0/10, 0%		0/10, 0%
tetA	Antibiotic efflux			8/10, 80%		3/10, 30%
tetC	Antibiotic efflux			0/10, 0%		2/10, 20%
YojI	Antibiotic efflux	13/14, 92.9%		**10/10, 100%**	2/10, 20%	**10/10, 100%**

QepA4	Antibiotic efflux	1/14, 7.14%		0/10, 0%		0/10, 0%
CTX-M-27	Antibiotic inactivation	3/14, 21.4%		0/10, 0%		0/10, 0%
CTX-M-123	Antibiotic inactivation	1/14, 7.14%		0/10, 0%		0/10, 0%
CTX-M-55	Antibiotic inactivation	2/14, 14.2%		5/10, 50%		9/10, 90%
CTX-M-15	Antibiotic inactivation	9/14, 64.2%		3/10, 30%		3/10, 30%
OXA-1	Antibiotic inactivation	0/14, 0%				
OXA-9	Antibiotic inactivation			0/10, 0%		2/10, 20%
TEM-1	Antibiotic inactivation	9/14, 64.2%		0/10, 0%		5/10, 50%
TEM-206	Antibiotic inactivation	1/14, 7.14%		5/10, 50%		5/10, 50%
TEM-215	Antibiotic inactivation					2/10, 20%
Escherichia coli ampC beta-lactamase	Antibiotic inactivation	1/14, 7.14%		0/10, 0%		0/10, 0%
DHA-1	Antibiotic inactivation	2/14, 14.2%		1/10, 10%		1/10, 10%
Mrx	Antibiotic inactivation	7/14, 50%	2/6, 33/3%	0/10, 0%		2/10, 20%
mphA	Antibiotic inactivation	7/14, 50%		0/10, 0%		9/10, 90%
AAC(3)-IId	Antibiotic inactivation	2/14, 14.2%		6/10, 60%		0/10, 0%
APH(6')-Ia	Antibiotic inactivation			1/10, 10%		
AAC(6')-Ib10	Antibiotic inactivation					7/10, 70%
APH(3')-Ia	Antibiotic inactivation			1/10, 10%		7/10, 70%
aadA	Antibiotic inactivation	3/14, 21.4%		4/10, 40%		9/10, 90%
aadA2	Antibiotic inactivation	4/14, 28.5%		0/10, 0%		5/10, 50%
aadA5	Antibiotic inactivation	1/14, 7.14%		0/10, 0%		6/10, 60%
aadA17	Antibiotic inactivation			0/10, 0%		2/10, 20%
TEM-135	Antibiotic inactivation			1/10, 10%		
aadA23	Antibiotic inactivation			0/10, 0%		5/10, 50%
linG	Antibiotic inactivation			0/10, 0%		2/10, 20%
dfrA12	Antibiotic target replacement	4/14, 28.5%		0/10, 0%		1/10, 10%
dfrA14	Antibiotic target replacement	4/14, 28.5%		5/10, 50%		7/10, 70%

dfrA17	Antibiotic target replacement		2/6, 33/3%			6/10, 60%
sul1	Antibiotic target replacement	5/14, 35.7%	2/6, 33/3%	0/10, 0%		1/10, 10%
sul2	Antibiotic target replacement	6/14, 42.8%		4/10, 40%		1/10, 10%
sul3	Antibiotic target replacement	3/14, 21.4%		6/10, 60%		9/10, 90%
dfrA15	Antibiotic target replacement			1/10, 10%		
bacA	Antibiotic target alteration	**14/14, 100%**		**10/10, 100%**	2/10, 20%	**10/10, 100%**
PmrF	Antibiotic target alteration	**14/14, 100%**		**10/10, 100%**	3/10, 30%	**10/10, 100%**
ArnT	Antibiotic target alteration	**14/14, 100%**		**10/10, 100%**		**10/10, 100%**
ugd	Antibiotic target alteration	8/14, 57.1%		9/10, 90%		0/10, 0%
eptA	Antibiotic target alteration	13/14, 92.9%		9/10, 90%		9/10, 90%
E448K	Antibiotic target alteration	12/14, 85.7%		**10/10, 100%**		7/10, 70%
E350Q	Antibiotic target alteration			9/10, 90%		
D350N, S357N	Antibiotic target alteration	13/14, 92.9%		**10/10, 100%**		**10/10, 100%**
D87N, S83L	Antibiotic target alteration			**10/10, 100%**		
R234F	Antibiotic target alteration	12/14, 85.7%		**10/10, 100%**		
S352T	Antibiotic target alteration	2/14, 14.2%				
S80I	Antibiotic target alteration			**10/10, 100%**		
Escherichia coli soxS with mutation conferring antibiotic resistance	Antibiotic target alteration; antibiotic efflux; reduced permeability	11/14, 78.5%		**10/10, 100%**		

Escherichia coli AcrAB-TolC with AcrR mutation conferring resistance to ciprofloxacin, tetracycline, and ceftazidime	Antibiotic target alteration; antibiotic efflux			**10/10, 100%**		
QnrB4	Antibiotic target protection	6/14, 42.8%		0/10, 0%		0/10, 0%
QnrB78	Antibiotic target protection	2/14, 14.2%		0/10, 0%		0/10, 0%
QnrS4	Antibiotic target protection	1/14, 7.14%		0/10, 0%		0/10, 0%
QnrS1	Antibiotic target protection	6/14, 42.8%		1/10, 10%		9/10, 90%
qnrB	Antibiotic target protection		2/6, 33/3%			
Antibiotic target alteration, antibiotic efflux	(Mechanism category)			**10/10, 100%**		

Prevalence shown as number of positive isolates / total isolates tested.
